# Sex-Specific Regulation of Gene Expression Networks by Surfactant Protein A (SP-A) Variants in Alveolar Macrophages in Response to *Klebsiella pneumoniae*

**DOI:** 10.3389/fimmu.2020.01290

**Published:** 2020-06-24

**Authors:** Nithyananda Thorenoor, Yuka Imamura Kawasawa, Chintan K. Gandhi, Joanna Floros

**Affiliations:** ^1^Center for Host Defense, Inflammation, and Lung Disease (CHILD) Research, Department of Pediatrics, The Pennsylvania State University College of Medicine, Hershey, PA, United States; ^2^Biochemistry & Molecular Biology, The Pennsylvania State University College of Medicine, Hershey, PA, United States; ^3^Pharmacology & Biochemistry & Molecular Biology, Institute for Personalized Medicine, The Pennsylvania State University College of Medicine, Hershey, PA, United States; ^4^Obstetrics & Gynecology, The Pennsylvania State University College of Medicine, Hershey, PA, United States

**Keywords:** surfactant protein A, surfactant protein-A1, surfactant protein-A2, alveolar macrophage, *Klebsiella pneumoniae*, TNF, TP-53, cell cycle signaling

## Abstract

Surfactant protein A (SP-A) in addition to its surfactant-related functions interacts with alveolar macrophages (AM), the guardian cells of innate immunity in the lungs, and regulates many of its functions under basal condition and in response to various pressures, such as infection and oxidative stress. The human SP-A locus consists of two functional genes, *SFTPA1* and *SFTPA2*, and one pseudogene. The functional genes encode human SP-A1 and SP-A2 proteins, respectively, and each has been identified with several genetic variants. SP-A variants differ in their ability to regulate lung function mechanics and survival in response to bacterial infection. Here, we investigated the effect of hSP-A variants on the AM gene expression profile in response to *Klebsiella pneumoniae* infection. We used four humanized transgenic (hTG) mice that each carried SP-A1 (6A^2^, 6A^4^) or SP-A2 (1A^0^, 1A^3^), and KO. AM gene expression profiling was performed after 6 h post-infection. We found: (a) significant sex differences in the expression of AM genes; (b) in response to infection, 858 (KO), 196 (6A^2^), 494 (6A^4^), 276 (1A^0^), and 397 (1A^3^) genes were identified (*P* < 0.05) and some of these were differentially expressed with ≥2 fold, specific to either males or females; (c) significant SP-A1 and SP-A2 variant-specific differences in AM gene expression; (d) via Ingenuity Pathway Analysis (IPA), key pathways and molecules were identified that had direct interaction with TP53, TNF, and cell cycle signaling nodes; (e) of the three pathways (TNF, TP-53, and cell cycle signaling nodes) studied here, all variants except SP-A2 (1A^3^) female, showed significance for at least 2 of these pathways, and KO male showed significance for all three pathways; (f) validation of key molecules exhibited variant-specific significant differences in the expression between sexes and a similarity in gene expression profile was observed between KO and SP-A1. These results reveal for the first time a large number of biologically relevant functional pathways influenced in a sex-specific manner by SP-A variants in response to infection. These data may assist in studying molecular mechanisms of SP-A-mediated AM gene regulation and potentially identify novel therapeutic targets for *K. pneumoniae* infection.

## Introduction

Bacterial mediated infectious lung diseases are an important worldwide cause of morbidity and mortality. *K. pneumoniae* is the leading bacterial cause of community and hospital-acquired respiratory infection (1, 2). It is an encapsulated gram-negative bacterium that resides in the environment such as in soil, surface waters and, on medical devices (3, 4). More importantly, *K. pneumoniae* colonizes in human mucosal surfaces, including the gastrointestinal tract and oropharynx (3–5). From these sites, it can gain entry to other tissues and cause a wide range of infections, e.g., pneumonia, urinary tract infections, bacteremia, and liver abscesses (6). Pulmonary infections caused by *K. pneumoniae* are particularly concerning as these are often characterized by a rapid clinical course, leaving a very short time for effective antibiotic treatment (7). This in turn results in high levels of morbidity and mortality. It has been observed that acute inflammatory responses (within hours of infection) include recruitment of neutrophils in the air spaces of the lungs and pulmonary edema in humans as well as in mice (8–10). Alveolar macrophages (AM) play a critical role in the clearance of bacteria from the lung through phagocytosis and depletion of AM resulting in a reduced killing of *K. pneumoniae in-vivo* (11). To control the infection an early inflammatory response occurs as AM produces inflammatory cytokines. These are essential for a rapid and effective immune response during the early stages of lung infection, as well as during the progression of infection (12, 13).

The effects of sex and sex hormones on pulmonary infection in humans and animals are well-established (14). Males typically exhibit weaker humoral and cell-mediated immune responses (15), and delayed lung maturation (16, 17) compared to females. It has also been observed that the number and the activity of cells involved in innate immunity differ between sexes (18, 19) as well as in lung diseases (20–24). Animal models of respiratory infection have shown that sex influences susceptibility, and severity of disease (25–32) and that sex hormones play a role (33). Therefore, it is important to identify and study the factors that can influence the incidence, susceptibility, and severity of lung diseases. Among them, innate host defense and sex are important contributing factors.

Pulmonary surfactant proteins, particularly the hydrophilic surfactant proteins (SPs), serve as a first line of contact for inhaled bacteria entering the lung and are thought to play a role in colonization and pathogenesis. SP-A is a member of the collectin family with an N-terminal collagen-like domain and a C-terminal carbohydrate recognition domain that recognizes and binds to debris, pathogens, and allergens (34, 35). Besides that, SP-A also influences multiple AM-mediated host defense functions such as chemotaxis (36), enhancement of phagocytosis and bacterial killing by macrophages (37), and proliferation of dendritic cells (38–40). Unlike in rodents and most mammals, the human SP-A genetic locus consists of two functional genes, *SFTPA1* and *SFTPA2*, and one pseudogene (41, 42) encoding SP-A1 and SP-A2 proteins, respectively, and each gene has been identified with several genetic and splice variants (41, 43, 44).

Several studies have identified differences between SP-A1 and SP-A2 in both qualitative (i.e., functional, biochemical, and/or structural) (45–48), and quantitative (regulatory) functions (46, 49–55). In particular, these include surfactant secretion modulation (46), cytokine production (56–58), and phagocytosis by AM (47, 48, 59). Moreover, differences in the structure and posttranslational modification of SP-A1 and SP-A2 have been observed (60). However, both SP-A1 and SP-A2 are required to make tubular myelin, an extracellular form of surfactant (52). It is of interest that the SP-A1 and SP-A2 variants differentially affect the AM proteomic expression profile, the AM actin cytoskeleton (61–63), the AM miRNome (64), as well as the miRNome of alveolar type 2 cells (65), and the biophysical properties and structure of surfactant (66). Despite, our knowledge of the diverse functions of SP-A1 and SP-A2, there are still gaps in our understanding of how SP-A influences host defense and the cell types it affects during lung infection, particularly AMs.

The AM is the primary effector cell for lung innate immunity and exhibits a unique phenotype (67) that is influenced by SP-A (29, 30, 68–70), although the extent of this effect is not fully understood. Previous work has demonstrated the importance of SP-A, among others, on AM expression and survival. After administration of a single exogenous dose of SP-A to SP-A-KO mice, the SP-A rescued AM proteome was more like that of wild type mice (71), and the survival after *K. pneumoniae* infection was improved significantly in the SP-A rescued KO mice (32). Moreover, SP-A2 variants enhance bacterial phagocytosis more effectively than SP-A1 variants (47, 48). Previous studies have shown sex differences in lung function and disease susceptibility (29, 31, 32, 72, 73), and in risk, incidence, and pathogenesis of various lung diseases (74, 75). Human and animal studies have shown an increased incidence of respiratory infections and severity of pneumonia in males (23, 27). Furthermore, sex-dependent survival was observed in wild type and SP-A-KO mice in response to *K. pneumoniae* infection, with females exhibiting higher survival compared to males, and that pattern reversed after oxidative stress (29), with females exhibiting lower survival compared to males. A differential effect of sex on AM proteome (62) as well as the AM miRNome of a single gene product (SP-A1 or SP-A2) or both gene products (SP-A1 and SP-A2, co-ex) in response to oxidative stress has been observed (64, 76). In addition, the SP-A1 and SP-A2 variants have been shown to play a crucial role in the differential outcome of airway function with significant sex- and variant-specific differences in both males and females in response to *K. pneumoniae* infection (73). However, the underlying mechanisms and the impact of SP-A1 and/or SP-A2 gene products on AM gene regulation in response to *K. pneumoniae* infection remain unknown.

In the present study, we hypothesized that genetic variants of SP-A differentially regulate AM gene expression in response to infection. Toward this hTG mice that carried SP-A1 (6A^2^, 6A^4^) or SP-A2 (1A^0^, 1A^3^), and KO are infected with *K. pneumoniae* and the gene expression profiling of AM was studied. We found: (a) significant differences in gene expression among variants, as well as sex differences; (b) Ingenuity Pathway Analysis (IPA) revealed key pathways and molecules involved in TP53, TNF, and cell cycle signaling nodes. All variants except SP-A2 (1A^3^) female, showed significance for at least 2 of the three pathways studied, and KO male showed significance for all three pathways; (c) validation of key molecules exhibited variant-specific significant differences in their expression between sexes, and a similarity in the gene expression profile of KO and SP-A1 mice was observed. These data may assist in studying molecular mechanisms of SP-A-mediated AM gene regulation and potentially identify novel therapeutic targets for *K. pneumoniae* infection.

## Methods

### Animals

All mice used in the present study were 12 weeks of age. In this study, we used humanized transgenic (hTG) mice that carried SP-A1 (6A^2^, 6A^4^), SP-A2 (1A^0^, 1A^3^) variant, as well as SP-A knockout (KO) mice. hTG mice were generated on the C57BL6/J SP-A (KO) background (52). The animals used in this study were maintained as described previously (32, 73). Briefly, the animals were raised and maintained under approved housing in a pathogen-free condition, at the Penn State College of Medicine animal facility. Both males and females were used in this study. The females were synchronized with regard to the estrous cycle as described previously (32, 73). A total of 86 mice (56 for gene expression analysis and 30 for qRT-PCR analysis) were used. The Penn State University College of Medicine Institutional Animal Care and Use Committee (IACUC) approved all procedures involving animals.

### Expression of SP-A1 and SP-A2 Variants: Western Blot Analysis

**Figure 1:**
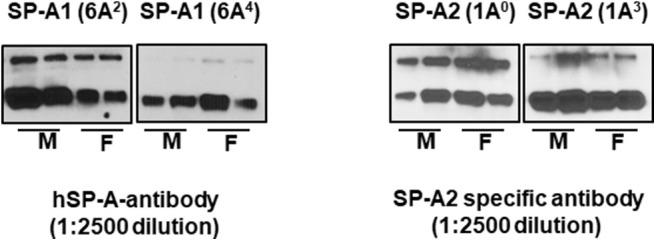
The expression levels of SP-A variants in BAL of males (M) and females (F) were monitored by western blot analysis. The hSP-A antibody (1:2500 dilution) recognizes both SP-A1 and SP-A2 proteins, whereas, the SP-A2 antibody (Aviva-ARP64034_P050, 1-2500 dilution) is specific to SP-A2 protein (no such antibody is available for SP-A1) and does not recognize SP-A1 proteins.

Equal amounts (20 μg) of bronchoalveolar lavage fluid from SP-A1 and SP-A2 hTG mice were separated by sodium dodecyl sulfate-polyacrylamide gel electrophoresis (SDS-PAGE), transferred to PVDF membrane, and the expression levels of SP-A variants (1A^0^, 1A^3^, 6A^2^, 6A^4^) were detected by Western blotting with specific antibodies. Samples from 2 males and 2 females were individually analyzed. Blots were incubated with hSP-A antibody (1:2500 dilution) recognizes both SP-A1 and SP-A2, whereas, the SP-A2 specific antibody (Aviva-ARP64034_P050, 1:2500 dilution) is specific to SP-A2 protein, and goat anti-rabbit (IgG) HRP-conjugated secondary antibody (1:5000 dilution) were used and detected by ECL method as shown in Figure 1.

### Preparation of Bacteria

*K. pneumoniae* bacteria (ATCC 43816) were obtained from American Tissue Culture Collection (Rockville, MD) and prepared as described previously (29, 30, 32, 73). Fifty μl of a suspension containing ~450 CFU were used to infect each mouse. The CFU/ml values were calculated based on the standard curve obtained at OD_660_ of the bacterial suspension.

### Infection of Mice With *K. pneumoniae*

The mice were anesthetized and infected with bacteria as described previously (29, 30, 32, 73, 77). Briefly, humanized transgenic (hTG) mice, SP-A1 (6A^2^, 6A^4^), SP-A2 (1A^0^, 1A^3^), and SP-A-KO male and female mice (*n* = 4/group) were anesthetized with a mixture of ketamine and xylazine and infected with *K. pneumoniae* (~450 CFU/mouse) in 50 μl of PBS oropharyngeal as described previously (77). The SP-A2 (1A^0^) mice were studied at a different time point (6, 18, and 24 h). Based on the finding of the SP-A2 (1A^0^) and previous studies (52, 71), we selected the 6 h time point for subsequent study of SP-A1 (6A^2^, 6A^4^), SP-A2 (1A^3^), and KO. Furthermore, we postulated that this time interval would allow us to study early AM gene expression changes in response to bacterial infection.

### Mouse Alveolar Macrophage Isolation

Mouse AM were obtained from both male and female mice by bronchoalveolar lavage (BAL) at 6, 18, and 24 h (SP-A2 (1A^0^) or 6 h after infection for SP-A1 (6A^2^, 6A^4^), SP-A2 (1A^3^) and KO (*n* = 4/group), as described previously (71). In brief, AMs were obtained by performing BAL with PBS containing 1 mM EDTA, using a volume equal to lung capacity (i.e., 6 times with 0.5 mL) a total of 3 ml. The fluid was instilled and withdrawn 3 times with chest massage during withdrawal, then centrifuged at 150 × g for 5 min at 4°C and the cell pellet washed with 1 mL of PBS containing, 1 mM EDTA. Cells were counted to obtain total cell counts (~2.5 × 10^6^ cells/mouse) before being frozen at −80°C for subsequent gene expression studies.

### RNA Preparation, Library Construction, and Sequencing

Total RNA was extracted using mirVana kit (#AM1560, Ambion, Waltham, MA). The extracted RNAs were quantified and quality checked using a BioAnalyzer RNA-6000 Pico Kit (Agilent Technologies, Santa Clara, CA) at the Penn State College of Medicine Genomic Core Facility. QuantSeq 3' mRNA-Seq Library Prep Kit FWD from Illumina (Lexogen, Vienna, Austria) was used to generate mRNA-Seq libraries as per manufacturer's recommendation, followed by deep sequencing on an Illumina HiSeq-2500 as per the manufacturer's instructions. Briefly, 0.5–1 ng of total RNA was subjected to the first cDNA strand which is initiated by oligo dT priming. The synthesis of the second cDNA strand is performed by random priming, in a manner that DNA polymerase is efficiently stopped when reaching the next hybridized random primer, so only the fragment closest to the 3′ end gets captured for later indexed adapter ligation and PCR amplification. The processed libraries were assessed for fragment size distribution and quantity using a BioAnalyzer High Sensitivity DNA kit (Agilent Technologies). Pooled libraries were denatured and loaded onto a TruSeq Rapid flow cell on an Illumina HiSeq 2500 (Illumina) and run for 50 cycles using a single-read recipe (TrueSeq SBS kit v3, Illumina) according to the manufacturer's instructions. Illumina CASAVA pipeline (released version 1.8, Illumina) was used to obtain de-multiplexed sequencing reads (fastq files) passed the default purify filter. These were further subjected to QuantSeq data analysis pipeline on a Bluebee genomics analysis platform (Bluebee, Cambridge, MA). The differentially expressed genes (DEG) between males and females were identified by using edgeR test method (78) and TCC v1.14.0 R package (79). The DEG are selected based on their fold change and their *P*-value (*P* < 0.05) for further analyses. We chose genes for further analysis based on their *P*-value (*P* < 0.05) and their expression levels (≥2-fold change) in AM cells from *K. pneumoniae* infected mice. The fold differences for the identified genes between males and females were determined by dividing a specific individual male gene value by the corresponding specific female gene value and vice versa for the same gene.

### Ingenuity Pathway Analysis

Ingenuity Pathway Analysis (IPA, www.qiagen.com/ingenuity Qiagen, Redwood City) was used to identify signaling network pathways and identify biological functions and regulatory networks of the differentially expressed genes in our experimental conditions. IPA analysis was performed on the genes that met the cut-off 2.0-fold up or downregulation in the male and female groups in the studied comparisons. Biological functions were identified via the Ingenuity Pathways Knowledge Base. We compared gene expression changes associated with the canonical pathway. We assessed the cellular and molecular classification of genes from the dataset with corresponding *P*-values (*P* < 0.05). All identified gene functions and networks are supported by the published literature in the Ingenuity Pathways Knowledge Base. The IPA software utilizes Fisher's exact test to obtain a *P*-value to identify the probability of association of the dataset to the canonical pathway, functions, and network associated with the dataset by assigning a score to the pathway.

### Real-Time PCR

For individual gene expression assays, SP-A1 (6A^2^, 6A^4^), SP-A2 (1A^0^, 1A^3^), and KO mice were infected with bacteria for 6 h and AMs were isolated as mentioned above. AMs were lysed by the addition of QIAzol Lysis Reagent (Qiagen, Germantown, MD). Total RNA was purified with Direct-zol RNA Mini-Prep kit (#R2052, Zymo Research, Irvine, CA), and RNA concentration was quantified by Nanodrop. RNA was reverse transcribed using RT2 first Stand kit (3220401, Qiagen), according to the manufacturer's protocol. The expression levels of ADAMTSL4, AKT1, AURKA, BCL3, BCL10, BTK, BUB1B, C1QC, CCL9, CCNA1, CCND1, CCRL2, CDK1, CDK2, CDKN1A, CDKN1B, CFLAR, CKAP2, CTTNB1, CXCL2, CXCR6, ERP44, ESPL1, FKBP5, FOSL1, GAPDH, GBP2, IER5, IFITM2, IRF1, KAT2B, LSP1, MARCO, MGMT, MMP12, MT2, MYC, MYD88, MYO1E, NKX3-1, PPARA, PPARG, PRDM1, PSMB8, RAG1, RCC2, RELA, RSAD2, RTP4, SAMHD1, STAT1, STAT3, STAT5a/b, TACC2, TAP1, TAP2, TCF4, TIE1, UHRF1, and ZFP36L1, were measured with real-time PCR with RT2 SYBR green ROX qPCR master mix (#330520, Qiagen) on a QuantStudio 12K Flex Real-Time PCR system (Applied Biosystems, Waltham, MA) at the Pennsylvanian State University College of Medicine Genomic Core Facility. AM cell samples from 3 mice/infection (males and females) in triplicates/animal were individually analyzed and quantified relative to GAPDH mRNA expression. The relative expression levels of genes were determined by the 2^−Δ*CT*^ method (ΔCT was calculated as follows: ΔCT = CT _gene−of−interest_ − CT _housekeepinggene_).

### Statistical Analysis

The statistical difference of the gene expression level in male compared to female and vice versa was evaluated by the two-tailed *t*-test and non-parametric Mann–Whitney *U*-test. All the data points are means ± standard deviation. All the analyses were performed using Graph-Pad Prism software version 5.0 (Graph-Pad Software, San Diego, USA). Values of *P* < 0.05 were considered statistically significant.

## Results

### Differential Expression of Genes in SP-A2 (1A^0^) Male and Female Mice Infected With *K. pneumoniae* and Studied at Different Time Points Post-Infection

**Figure 2:**
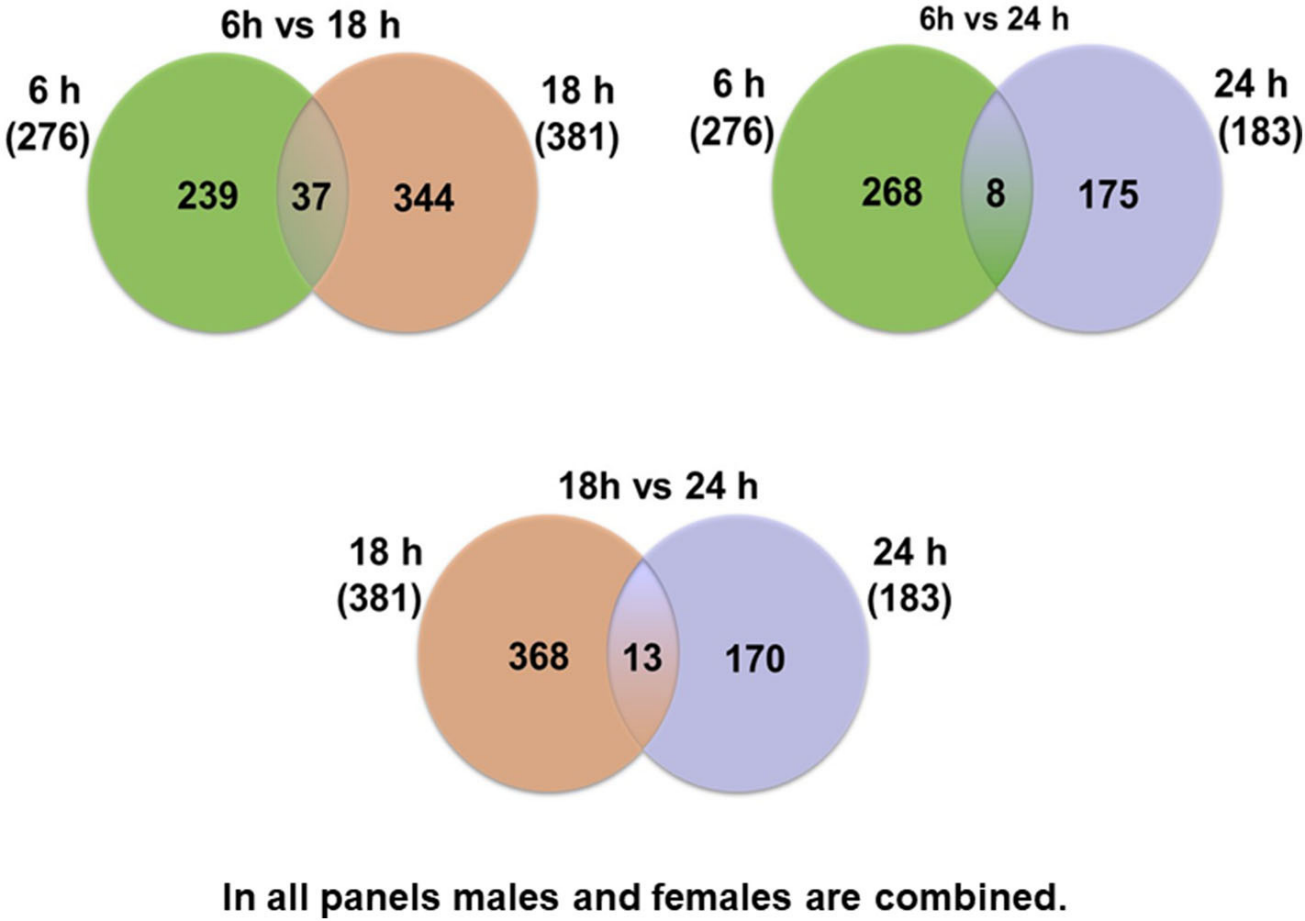
Comparison of the number of genes affected by *K. pneumoniae* in males and females at different time points. The Venn diagrams show genes identified in common after comparison of two different time points. Thirty-seven genes were in common between 6 vs. 18 h, and 239 were unique for the 6 h time point and 344 were unique for the 18 h time point. Eight genes were identified in common between 6 vs. 24 h, and 268 were unique for the 6 h time point and 175 were unique for the 24 h time point. The comparisons between 18 vs. 24 h revealed 13 genes in common in both groups and 368 unique for the 18 h and 170 unique for the 24 h time point.

At first, the expression of genes from AMs of SP-A2 (1A^0^) male and female mice was analyzed to identify differentially expressed genes in response to infection at different time points (6, 18, and 24 h). We identified, 276 genes (after 6 h infection), 381 genes (after 18 h infection), and 183 genes (after 24 h infection) differentially expressed with a *P*-value <0.05 in both male and female SP-A2 (1A^0^) mice (Supplementary File 1). To identify specific gene expression changes in response to infection, we compared the expression levels of genes significantly either increase (≥2-fold) or decrease (≤2-fold) in males compared females (M/F) and vice versa at different time points. Out of 276 genes identified after 6 h infection, the expression of 169 genes significantly increased ≥2-fold and 75 genes significantly decreased ≤ 2-fold in males compared to females, and vice versa (Table 1, Supplementary File 1). We identified, a total of 32 genes that had expression value between > 0.5 – <2-fold change either increase or decrease in males compared to females and vice versa (Table 1, Supplementary File 1). At the 18 h infected mice, out of 381 genes, 146 and 148 genes had expression levels ≥2-fold or ≤2-fold, respectively, in males compared to females, and vice versa (Table 1, Supplementary File 1). A total of 87 genes identified after 18 h post-infection had expression value between > 0.5–2-fold change either increase or decrease in males compared to females and vice versa (Table 1, Supplementary File 1). Whereas, at the 24 h post-infection, out of 183 genes, 79 and 66 genes had expression levels ≥2-fold or ≤ 2-fold, respectively in males compared to females, and vice versa (Table 1, Supplementary File 1). A total of 38 genes identified after 24 h post-infection had expression value between > 0.5 – <2-fold change either increase or decrease in males compared females and vice versa (Table 1, Supplementary File 1). Next, we compared genes at two different time points in males and females (≥2-fold or ≤ 2-fold, males compared to females and vice versa), and found 37 genes to be in common at 6 and 18 h post-infection (Figure 2, Table 2), 8 genes were in common at 6 and 24 h post-infection (Figure 2, Table 3), and 13 genes were in common at 18 and 24 h post-infection (Figure 2, Table 4). The Hba-a1 (hemoglobin alpha, adult chain 1) gene is identified to be in common in all the time points studied in response to infection (Tables 2–4). Since the 6 h time point resulted in significant differences in AM gene expression and also based on previous findings (52, 71), we focused our subsequent study for the other SP-A variants i.e., SP-A1 (6A^2^, 6A^4^), SP-A2 (1A^3^) and KO on the 6 h time point in order to identify early-stage gene expression changes after infection.

**Table 1:** The total number of genes identified with ≥2-fold change at different time points (6, 18, and 24 h) in SP-A2 (1A^0^) males compared to females after *K. pneumoniae* infection are shown.

	**SP-A2 (1A**^****0****^**)** **Male vs. female**	
**Gene variant # of genes identified**
	**≥2-fold change (increase)**	**≤2-fold change (decrease)**
6 h (*n* = 276)	169* (22)	75* (10)
18 h (*n* = 381)	146* (28)	148* (59)
24 h (*n* = 183)	79* (20)	66* (18)

* *Number of genes significantly changed ≥2-fold either increase or decrease in males compared to females (M/F). In parenthesis, genes that had expression value between > 0.5 – <2-fold change either increase or decrease in males compared females (M/F) are shown. All comparisons with or without the cut-off value had P-value < 0.05*.

**Table 2:** Relative expression levels of genes identified in common that changed ≥2-fold increased or ≤ 2-fold decreased at the 6 and 18 h time points in SP-A2 (1A^0^) males compared to females (M/F), and vice vera after *K. pneumoniae* infection are shown.

**Gene symbol**	**6 h**			**18 h**		
	**M vs. F**	**F vs. M**	***P*-value**	**M vs. F**	**F vs. M**	***P*-value**
Marcks	5.7460	0.1740	0.000089	2.7000	0.3704	0.001748
Btg2	4.0105	0.2493	0.000236	3.1590	0.3166	0.030556
Hcar2	3.9256	0.2547	0.000251	4.0331	0.2479	0.000005
S100a6	4.3518	0.2298	0.000370	3.3733	0.2964	0.000000
Gadd45b	5.6437	0.1772	0.000546	3.0560	0.3272	0.017165
Zfp36	5.0443	0.1982	0.000623	3.6399	0.2747	0.001794
Cd14	4.5731	0.2187	0.001439	4.2935	0.2329	0.011648
Ptafr	3.8673	0.2586	0.001631	4.2266	0.2366	0.000202
Junb	2.8988	0.3450	0.004945	2.6903	0.3717	0.003759
Slc25a37	3.4459	0.2902	0.004990	2.6604	0.3759	0.022788
Cd2	0.3728	2.6824	0.005315	0.4408	2.2688	0.022468
Hba-a1*	9.6062	0.1041	0.005981	0.4338	2.3052	0.015265
RP23-59N15.4	5.0270	0.1989	0.006044	5.9803	0.1672	0.000588
mt-Ta	2.6127	0.3827	0.006904	3.5170	0.2843	0.001029
mt-Tq	3.0148	0.3317	0.007714	1.9598	0.5103	0.020162
Snx20	2.2940	0.4359	0.007715	2.2880	0.4371	0.021775
Trib1	2.7831	0.3593	0.008952	2.3894	0.4185	0.009813
Clec4d	3.3064	0.3024	0.009817	4.1494	0.2410	0.002481
Zfp36l1	2.9784	0.3357	0.010499	2.9442	0.3397	0.001794
Mbp	0.3419	2.9252	0.010910	3.1897	0.3135	0.024033
Cmtm7	3.0588	0.3269	0.011060	2.7641	0.3618	0.030862
Gys1	2.6634	0.3755	0.013845	0.3495	2.8612	0.024641
1200014J11Rik	0.4144	2.4132	0.017435	2.4349	0.4107	0.023370
Gdf15	1.8999	0.5263	0.020232	2.6002	0.3846	0.002174
Ier5	2.0183	0.4955	0.021404	1.9254	0.5194	0.045583
Arl4c	2.9374	0.3404	0.021631	2.5632	0.3901	0.013733
Lck	4.6324	0.2159	0.028625	4.5117	0.2216	0.000796
S100a8	8.7843	0.1138	0.030529	3.5413	0.2824	0.000181
Gga1	1.9733	0.5068	0.032244	2.2715	0.4402	0.043944
Mxd1	5.2531	0.1904	0.032654	3.0207	0.3311	0.000770
Clec5a	2.3036	0.4341	0.034274	0.3721	2.6876	0.011033
Ccr1	2.3035	0.4341	0.037209	2.1519	0.4647	0.048452
mt-Tm	1.9731	0.5068	0.038017	1.9757	0.5061	0.029326
Adora2a	2.7494	0.3637	0.040193	3.8980	0.2565	0.006020
Ier3	4.7039	0.2126	0.042962	2.2038	0.4538	0.001985
Hif1a	2.5693	0.3892	0.044278	2.0839	0.4799	0.022312
Tbkbp1	2.2167	0.4511	0.049618	2.2612	0.4422	0.036860

* *Identified in all studied time points*.

**Table 3:** Relative expression levels of genes identified in common that changed ≥2-fold increased or ≤ 2-fold decreased at the 6 and 24 h time points in SP-A2 (1A^0^) males compared to females (M/F), and vice versa after *K. pneumoniae* infection are shown.

**Gene symbol**	**6 h**			**24 h**		
	**M vs. F**	**F vs. M**	***P*-value**	**M vs. F**	**F vs. M**	***P*-value**
Hba-a2	7.5810	0.1319	0.03314	2.65	0.3770	0.0055
Rnf213	2.8878	0.3463	0.04376	0.27	3.6470	0.0087
Ifi203	3.5553	0.2813	0.00288	0.31	3.2390	0.0088
Nod1	2.2943	0.4359	0.02315	0.49	2.0484	0.0149
Mt2	0.4431	2.2569	0.01686	0.52	1.9203	0.0302
Rab44	3.2471	0.3080	0.00166	0.55	1.8026	0.0333
Samd9l	5.0088	0.1996	0.00234	0.44	2.2910	0.0379
Hba-a1*	9.6062	0.1041	0.00598	4.01	0.2492	0.0001

* *Identified in all studied time points*.

**Table 4:** Relative expression levels of genes identified in common that changed ≥2-fold increased or ≤ 2-fold decreased at the 18 and 24 h time points in SP-A2 (1A^0^) males compared to females (M/F), and vice versa after *K. pneumoniae* infection are shown.

**Gene symbol**	**18 h**			**24 h**		
	**M vs. F**	**F vs. M**	***P*-value**	**M vs. F**	**F vs. M**	***P*-value**
Bst2	2.0864	0.4793	0.03311	0.3552	2.8152	0.00195
Tor1b	0.3214	3.1113	0.00297	3.3859	0.2953	0.00246
Lrrc17	2.2032	0.4539	0.02549	0.4665	2.1434	0.00537
Gba	0.4121	2.4266	0.0142	2.2229	0.4499	0.00619
mt-Nd6	3.2300	0.3096	0.03552	0.3490	2.8652	0.0078
Gm8995	2.2180	0.4509	0.01838	0.3575	2.7969	0.00982
mt-Tv	3.5025	0.2855	0.00848	0.4122	2.4263	0.02662
Irf1	3.2370	0.3089	0.00919	0.3665	2.7285	0.02888
Siae	0.2506	3.9905	0.00666	3.1088	0.3217	0.0351
Ndufs8	0.3328	3.0050	0.01062	1.8580	0.5382	0.03568
Rpl37a	0.6047	1.6538	0.03723	1.5528	0.6440	0.03766
Hba-a1*	0.4338	2.3052	0.01526	4.0131	0.2492	0.00014
Tgs1	0.3697	2.7051	0.02498	1.6874	0.5926	0.04624

* *Identified in all studied time points*.

### Sex Differences in AMs Gene Expression in Response to *K. pneumoniae*

**Figure 3:**
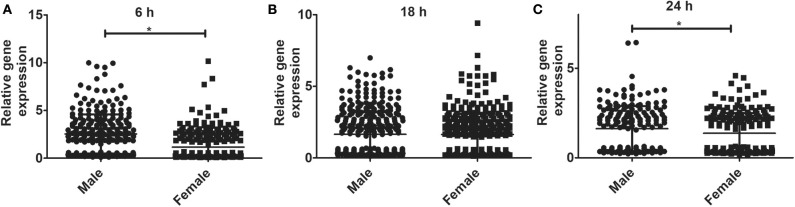
Gene expression in AMs of SP-A2 (1A^0^) male and female after infection with *K. pneumoniae*. Comparisons between genes identified in males and females after 6 h (276 genes), 18 h (381 genes), and 24 h (183 genes) of infection (*n* = 4/group). Significant differences were observed in the expression of genes at the 6 h and 24 h time points between males and females **(A,C)**, with no significant differences at the 18 h time point **(B)**. Significant differences *P* < 0.05 between sexes are noted with an asterisk (*).

**Figure 4:**
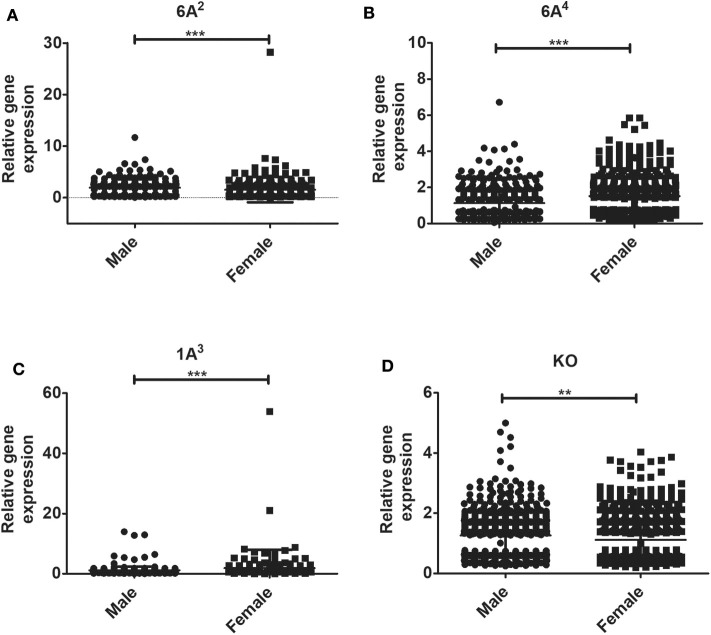
Gene expression in AMs of SP-A1 (6A^2^, 6A^4^) and SP-A2 (1A^3^) variants as well as KO male and female after *K. pneumoniae* infection at the 6 h time point. A total of 196 genes (SP-A1 (6A^2^), **(A)**, 494 genes (SP-A1 (6A^4^), **(B)**, 397 genes (SP-A2 (1A^3^), **(C)** and 858 genes (KO, **D**) were identified with a *P*-value < 0.05 (*n* = 4/group). Significant sex differences in the expression of genes were observed for all variants studied and the KO. The significant differences (*P* < 0.01 and *P* < 0.001) between sexes are noted with an asterisk (** and ***).

The relative expression levels of the significant genes (*P* < 0.05) in the AMs of male vs. female mice after infection were analyzed. The two-tailed *t*-test and non-parametric Mann–Whitney *U-*test resulted in significant sex difference between genes identified from males and females at the 6 h, and 24 h time point (Figures 3A,C), whereas the genes identified at 18 h (Figure 3B) time point did not have any significant difference between males and females of SP-A2 (1A^0^). A similar analysis resulted in significant sex differences in the expression of AM genes in other SP-A variants, i.e., SP-A1 (6A^2^, 6A^4^), SP-A2 (1A^3^), as well as in KO males and females at 6 h post-infection (Figures 4A-D).

### Differential Expression of Genes in SP-A1 (6A^2^, 6A^4^), SP-A2 (1A^3^) and KO Males and Females Mice Infected With *K. pneumoniae*

In response to infection, we identified 196 genes [SP-A1 (6A^2^)], 494 genes [SP-A1 (6A^4^)], 397 genes (SP-A2 (1A^3^)] and 858 genes (KO) with a *P*-value < 0.05 in both males and females (Supplementary File 2). Out of 196 genes identified from AMs from SP-A1 (6A^2^) after infection, 97 and 61 genes had expression levels ≥2-fold or ≤2-fold, respectively, in males compared to females, and vice versa (Table 5, Supplementary File 2). Thirty-eight genes had expression value between >0.5 – <2-fold change either increase or decrease in males compared to females and vice versa (Table 5, Supplementary File 2). In the case of SP-A1 (6A^4^), 494 genes were identified, 54 and 119 genes had expression levels ≥2-fold or ≤2-fold in males compared to females, and vice versa (Table 5, Supplementary File 2). The levels of 321 genes had expression value between >0.5 – <2-fold change either increase or decrease in males compared to females and vice versa (Table 5, Supplementary File 2).

**Table 5:** The total number of genes identified from SP-A1 and SP-A2 variants and KO males and females with ≥2-fold change after *K. pneumoniae* infection (6 h) are shown.

**Gene variant and # of genes identified**	**Male vs. female**	
	**≥ 2-fold change (increase)**	**≤ 2-fold change (decrease)**
SP-A2 (1A^0^) (*n* = 276)	169* (22)	75* (10)
SP-A1 (6A^2^) (*n* = 196)	97* (18)	61* (20)
SP-A1 (6A^4^) (*n* = 494)	54* (187)	119* (134)
SP-A2 (1A^3^) (*n* = 397)	11* (204)	62* (120)
KO (*n* = 858)	93* (416)	90* (259)

* *Number of genes significantly changed ≥2-fold either increase or decrease in males compared to females (M/F). In parenthesis, genes that had expression value between >0.5 – <2-fold change either increase or decrease in males compared to females (M/F) are shown. All comparisons with or without the cut-off value had P-value <0.05*.

In a similar analysis with SP-A2 (1A^3^), 397 genes were identified, 11 and 62 genes had expression level ≥2-fold or ≤2-fold, respectively, in males compared to females, and vice versa (Table 5, Supplementary File 2). The levels of 324 genes had expression value between >0.5 – <2-fold change either increase or decrease in males compared females, and vice versa (Table 5, Supplementary File 2). Whereas, in KO, 858 genes were identified, and 93 and 90 genes had their expression level ≥2-fold or ≤2-fold, respectively, in males compared to females and vice versa (Table 5, Supplementary File 2). The levels of 675 genes had expression value between >0.5 – <2-fold change either increase or decrease in males compared females and vice versa (Table 5, Supplementary File 2).

### Differences in Gene Expression Between Gene-Specific Variants in Response to Infection

#### SP-A1

The bacterial infection resulted in significant differences in AM gene expression between males and females of 6A^2^ and 6A^4^ variants. Five genes with significantly changed levels were found to be in common between SP-A1 (6A^2^ vs. 6A^4^) variants in both males and females (Supplementary Figure 1A, Table 6), 191 (out of 196) genes are specific to 6A^2^, and 489 (out of 494) are specific to 6A^4^ (Supplementary File 3).

**Table 6:** Genes identified to be in common in males and females of SP-A1 (6A^2^ vs. 6A^4^) and their expression levels in response to infection (post 6 h).

**Gene symbol**	**SP-A1 (6A**^****2****^**)**			**SP-A1 (6A**^****4****^**)**		
	**M vs. F**	**F vs. M**	***P*-value**	**M vs. F**	**F vs. M**	***P*-value**
Xist	0.3690	2.7099	0.000001	0.7587	1.3181	0.000001
Fbxw5	5.0250	0.1990	0.000198	1.6996	0.5884	0.008145
Socs3	0.3991	2.5059	0.019144	0.4135	2.4185	0.023588
Fkbp5	0.4209	2.3760	0.021115	2.0533	0.4870	0.021180
Ikbkb	1.8006	0.5554	0.036103	1.3621	0.7342	0.020937

*The expression levels of all the genes shown above are significantly changed either increased ≥2-fold or decreased ≤2-fold in males compared to females (M/F), and vice versa*.

#### SP-A2

In response to infection in 1A^0^ and 1A^3^, significant changes in gene expression between the variants were observed. Thirty-one genes with significantly changed levels were found to be in common between SP-A2 (1A^0^ vs. 1A^3^) variants in both males and females (Supplementary Figure 1B, Table 7), and 245 (out of 276) genes are specific to 1A^0^ and 366 (out of 397) are specific to 1A^3^ (Supplementary File 3).

**Table 7:** Genes identified to be in common in males and females of SP-A2 (1A^0^ vs. 1A^3^) and their expression levels in response to infection (post 6 h).

**Gene symbol**	**SP-A2 (1A**^****0****^**)**			**SP-A2 (1A**^****3****^**)**		
	**M vs. F**	**F vs. M**	***P*-value**	**M vs. F**	**F vs. M**	***P*-value**
Marcks	5.7460	0.1740	0.000089	0.1409	7.0953	0.000387
Hcar2	3.9256	0.2547	0.000251	0.5103	1.9598	0.029532
Mmp8	5.8419	0.1712	0.000283	0.3390	2.9497	0.000347
Cd52	2.4022	0.4163	0.000366	0.6767	1.4778	0.032191
Ets2	4.1299	0.2421	0.000594	0.3624	2.7591	0.008474
Zfp36	5.0443	0.1982	0.000623	0.2009	4.9779	0.000001
Il1b	7.0992	0.1409	0.001554	0.1858	5.3821	0.003893
Ptafr	3.8673	0.2586	0.001631	0.3820	2.6177	0.044783
Grina	2.9707	0.3366	0.003259	0.6000	1.6665	0.017180
Btg1	2.7333	0.3659	0.003423	0.6250	1.5999	0.035135
Junb	2.8988	0.3450	0.004945	0.4618	2.1655	0.013529
Hba-a1	9.6062	0.1041	0.005981	13.9534	0.0717	0.000160
Bub1b	0.3247	3.0802	0.006720	1.3657	0.7322	0.037102
C3	2.5675	0.3895	0.007019	0.4196	2.3832	0.000217
Slco2b1	0.2867	3.4882	0.007774	1.5336	0.6521	0.019160
Irg1	7.1090	0.1407	0.009863	0.1385	7.2226	0.021020
Cdh1	0.4963	2.0150	0.012214	0.5022	1.9911	0.023057
Dgat1	3.4779	0.2875	0.016083	0.4271	2.3415	0.003079
Neurl1b	0.4998	2.0007	0.016627	1.4055	0.7115	0.006639
Gdf15	1.8999	0.5263	0.020232	0.3664	2.7290	0.000576
Ncor2	0.4623	2.1629	0.021449	1.5294	0.6539	0.004735
Tap1	7.0453	0.1419	0.021486	0.3311	3.0204	0.000002
Fcgr3	2.0423	0.4896	0.025231	0.6300	1.5872	0.022757
Krt79	0.5853	1.7086	0.029096	1.4907	0.6708	0.004495
S100a8	8.7843	0.1138	0.030529	0.1406	7.1117	0.014119
Gga1	1.9733	0.5068	0.032244	0.5886	1.6991	0.002916
Hba-a2	7.5810	0.1319	0.033145	12.9415	0.0773	0.000299
S100a9	9.9365	0.1006	0.034441	0.1133	8.8224	0.025052
Slc7a11	2.3295	0.4293	0.035834	0.2750	3.6359	0.002163
Vimp	0.4725	2.1162	0.038036	0.6624	1.5095	0.026797
Ier3	4.7039	0.2126	0.042962	0.2160	4.6307	0.004553

*The expression levels of all the genes shown above are significantly changed either increase ≥2-fold or decrease ≤2-fold in males compared to females (M/F), and vice versa*.

### Differences Between SP-A1 and SP-A2 variants in response to infection

The SP-A2 (1A^0^) males and females exhibited significant changes in the expression of AM genes (*n* = 276) in response to infection compared to SP-A1 (6A^2^) genes (*n* = 196). Ten genes were identified to be in common (Supplementary Figure 1C, Supplementary File 3), with 266 genes being specific to 1A^0^ and 186 to 6A^2^. A similar comparison between 1A^0^ vs. 6A^4^ resulted in the identification of 276 vs. 494 genes, respectively, with 15 genes being in common between variants (Supplementary Figure 1D, Supplementary File 3), and 261 genes were specific to 1A^0^ and 479 genes to 6A^4^.

The SP-A2 (1A^3^), males and females, exhibited significant changes in the expression of AM genes in response to infection compared to SP-A1 (6A^2^) (397 and 196, respectively). Fourteen genes were identified in common in both variants (Supplementary Figure 1E, Supplementary File 3), and 384 genes were specific to 1A^3^ and 182 genes to 6A^2^. A similar comparison between 1A^3^ vs. 6A^4^ resulted in the identification of 397 and 494 genes, respectively. Thirty-one genes were identified in common between variants (Supplementary Figure 1F, Supplementary File 3), and 366 genes were specific to 1A^3^ and 463 genes to 6A^4^.

### Differences Between KO and SP-A1 or SP-A2 Variants

The KO exhibited significant changes in the expression of genes in response to infection in males and females compared to SP-A variants as shown in Supplementary Figures 2A–D. From the AMs of KO males and females, a total of 858 genes were identified. Out of 858 genes 40, 142, 27, and 42 genes were identified in common to 1A^0^, 1A^3^, 6A^2^, and 6A^4^, respectively (Supplementary Figures 2A–D, Supplementary File 3).

### Ingenuity Pathway Analysis and Validation of the Expression of Key Molecules

To understand and integrate the AM gene expression, IPA was performed for genes whose expression was significantly altered ≥2-fold by *K. pneumoniae* infection, at the 6 h time point between males and females, from SP-A1 (6A^2^, 6A^4^), SP-A2 (1A^0^, 1A^3^), and KO mice.

**Figure 5:**
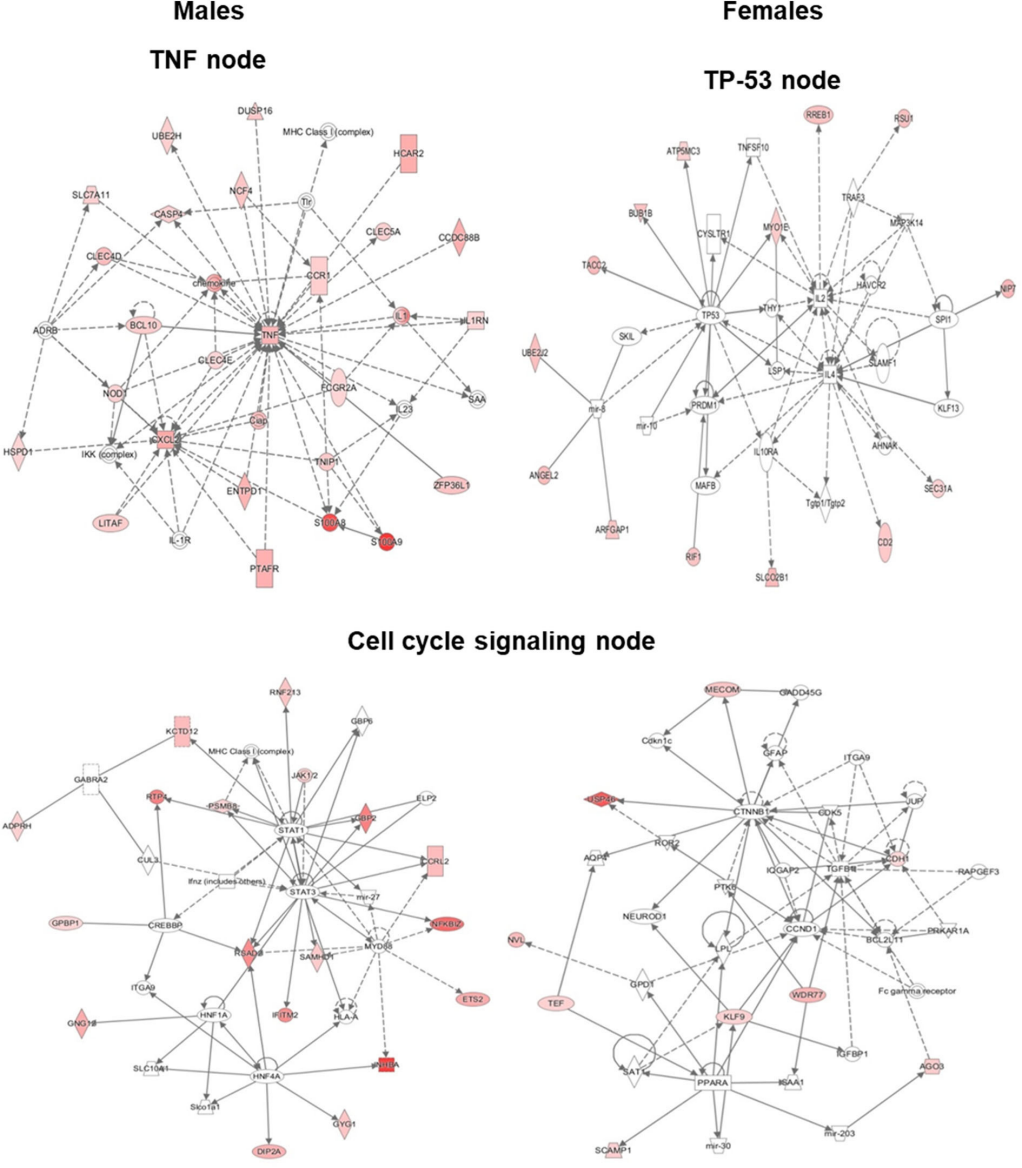
Ingenuity pathway analysis (IPA). The diagram depicts biological networks of genes whose expression was ≥2-fold (shown in color) in the AMs of SP-A2 (1A^0^) males vs. females at the 6 h post-infection time point. The diagram shows reported direct (solid lines) and indirect (dashed lines) interactions for these genes. (**Left**) genes and pathways in male mice; (**Right**) genes and pathways in female mice. Each gene or group of genes is represented as a node. Molecules that are significantly altered ≥2 are represented as node in red. Node shapes represent functional classes of gene products: Square for cytokines, Concentric (double) circle for complex/group, Diamonds for enzymes and peptidases, Ovals for transcription regulators and transmembrane receptors, Triangle for phosphatases and kinases, Rectangles for ligand-dependent nuclear receptors, G-protein coupled receptors, and ion channels, Trapezoids for transporters and microRNAs.

Based on the IPA results, we subsequently studied key pathways and found that the TP53, TNF, and cell cycle signaling nodes had direct interaction with 4 or more molecules in at least one of the studied variants. Although the TNF node in SP-A2 (1A^0^) males did not have 4 or more direct interactions, large number of genes (40–70% higher than any other node) with ≥2-fold change had indirect interaction with TNF node. Our subsequent analysis and gene validation were focused on molecules involved in these pathways. The functional relationship plots for SP-A2 (1A^0^) males and females are shown in Figure 5, and these indicate that many of the genes that had expression levels ≥2-fold in males vs. females have been reported to have direct (solid lines) or indirect (dashed lines) associations with TP53 (females), TNF (males), and cell cycle signaling nodes (males and females). A similar analysis of the genes identified, from males and females of SP-A1 (6A^2^, 6A^4^), SP-A2 (1A^3^) variants, and KO, whose expression was ≥2-fold in response to infection also showed the association of genes with TP53, TNF, and cell cycle signaling nodes with direct (solid lines) and indirect (dashed lines) (Supplementary Figure 3) indicating that among SP-A variants these pathways may be differentially activated and to a varying degree in response to infection.

The cell cycle signaling node was significant (*P* < 0.05) in SP-A2 (1A^0^) males and females, SP-A1 (6A^2^) males, SP-A2 (1A^3^) females, and KO males (Figure 5, Supplementary Figure 3). The TP-53 node (*P* < 0.05) was significant in both males and females in SP-A1 (6A^2^ and 6A^4^) and KO, but only in females in SP-A2 (1A^0^) (Figure 5, Supplementary Figure 3). The TNF node (*P* < 0.05) was significant only in SP-A2 (1A^0^) males and KO males (Figure 5, Supplementary Figure 3). None of the TP-53, TNF, or cell cycle nodes were significant in SP-A2 (1A^3^) males or any other pathways (Figure 5, Supplementary Figure 3).

From these pathways, we selected the following genes for further analysis (i.e., validation), ADAMTSL4, AKT1, AURKA, BCL3, BCL10, BTK, BUB1B, C1QC, CCL9, CCNA1, CCND1, CCRL2, CDK1, CDK2, CDKN1A, CDKN1B, CFLAR, CKAP2, CTTNB1, CXCL2, CXCR6, ERP44, ESPL1, FKBP5, FOSL1, GBP2, IER5, IFITM2, IRF1, KAT2B, LSP1, MARCO, MGMT, MMP12, MT2, MYC, MYD88, MYO1E, NKX3-1, PPARA, PPARG, PRDM1, PSMB8, RAG1, RCC2, RELA, RSAD2, RTP4, SAMHD1, STAT1, STAT3, STAT5a/b, TACC2, TAP1, TAP2, TCF4, TIE1, UHRF1, and ZFP36L1 (Figure 5, Supplementary Figure 3). The expression of the selected set of genes was evaluated by qRT-PCR from AMs of SP-A1 (6A^2^, 6A^4^), SP-A2 (1A^0^, 1A^3^), and KO male and female mice exposed to infection.

#### TNF-Node (Pro-Inflammatory Responses)

**Figure 6:**
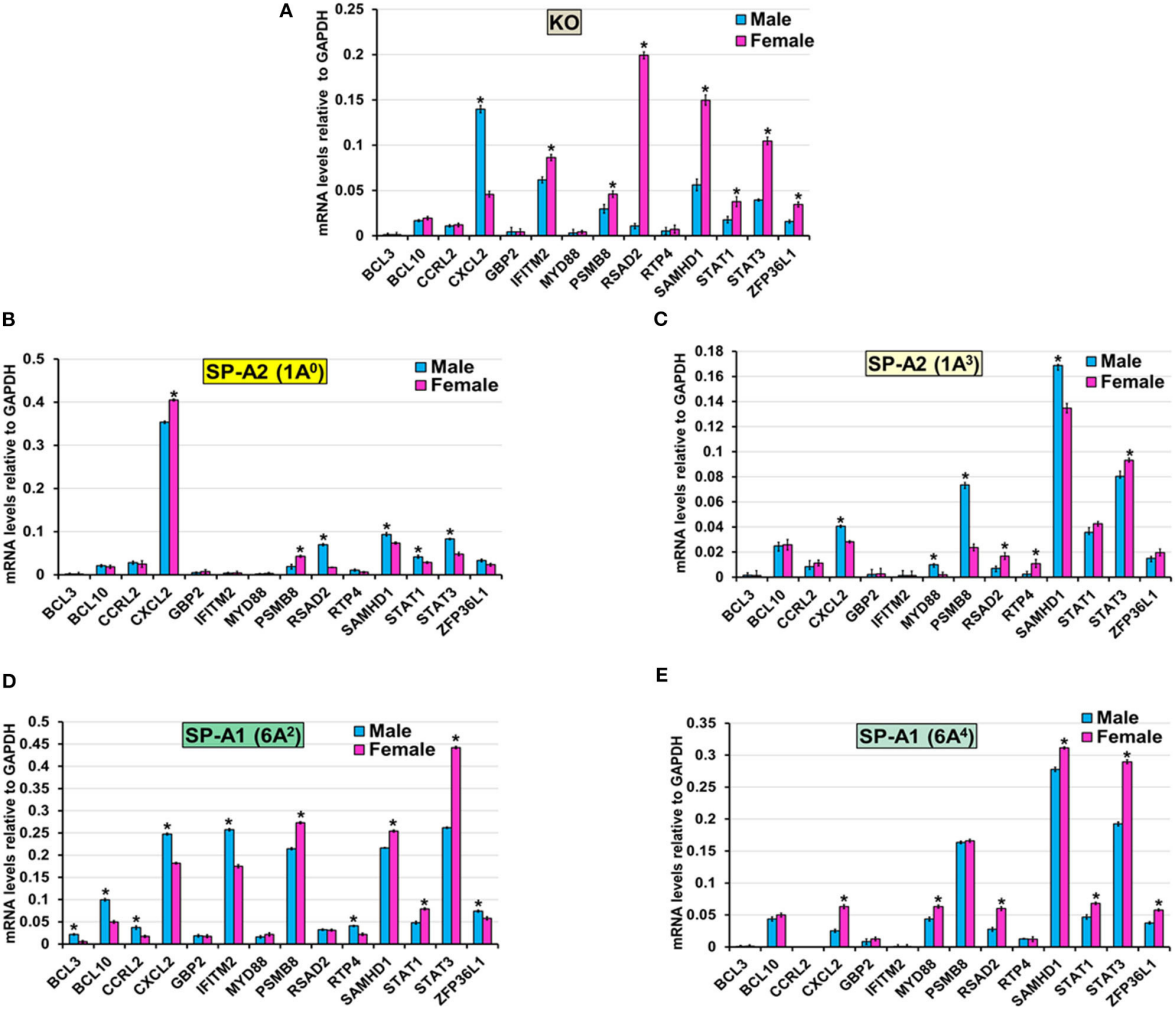
Effect of infection on the expression of genes involved in the TNF-node in SP-A1 (6A^2^, 6A^4^), SP-A2 (1A^0^, 1A^3^), and KO male and female. The expression level of genes involved in direct and indirect interactions with TNF shown in Figure 5A and Supplementary Figure 3 (BCL3, BCL10, CCRL2, CXCL2, GBP2, IFITM2, MYD88, PSMB8, RSAD2, RTP4, SAMHD1, STAT1, STAT3, and ZFP36L1) were measured in KO **(A)**, SP-A2 (1A^0^) **(B)**, SP-A2 (1A^3^) **(C)**, SP-A1 (6A^2^) **(D)** and SP-A1 (6A^4^) **(E)**. The expression levels were normalized to GAPDH and the significant differences (*P* < 0.05) between sexes are noted with an asterisk (*).

The expression level of BCL3, BCL10, and CCRL2 (6A^4^, 1A^0^, 1A^3^, and KO), GBP2 (6A^2^, 6A^4^, 1A^0^, 1A^3^, and KO), MYD88 (KO, 1A^0^, and 6A^2^), RTP4 (KO, 1A^0^, and 6A^4^), IFITM2 (1A^0^, 1A^3^, and 6A^4^), ZFP36L1 (1A^0^, 1A^3^), STAT1 (1A^3^), RSAD2 (6A^2^), and PSMB8 (6A^4^) (Figure 6) was similar between males and females in response to infection. However, in response to infection, male mice exhibited higher expression levels of CXCL2 (KO, 1A^3^, and 6A^2^), SAMHD1 (1A^0^, 1A^3^), RSAD2, STAT1, and STAT3 (1A^0^), MYD88 and PSMB8 (1A^3^), BCL3, BCL10, CCRL2, IFITM2, RTP4, and ZFP36L1 (6A^2^), compared to females (Figure 6). The female mice exhibited higher expression levels of CXCL2 (1A^0^, 6A^4^), PSMB8 (KO, 1A^0^, and 6A^2^), IFITM2 (KO), MYD88 (6A^4^), RSAD2 (KO, 6A^4^, and 1A^3^), SAMHD1 (KO, 6A^2^, and 6A^4^), STAT1 (KO, 6A^2^, and 6A^4^), STAT3 (KO, 6A^2^, 6A^4^, and 1A^3^), ZFP36L1 (KO, 6A^4^), and RTP4 (1A^3^) compared to males in response to infection (Figure 6).

#### TP-53-Node

**Figure 7:**
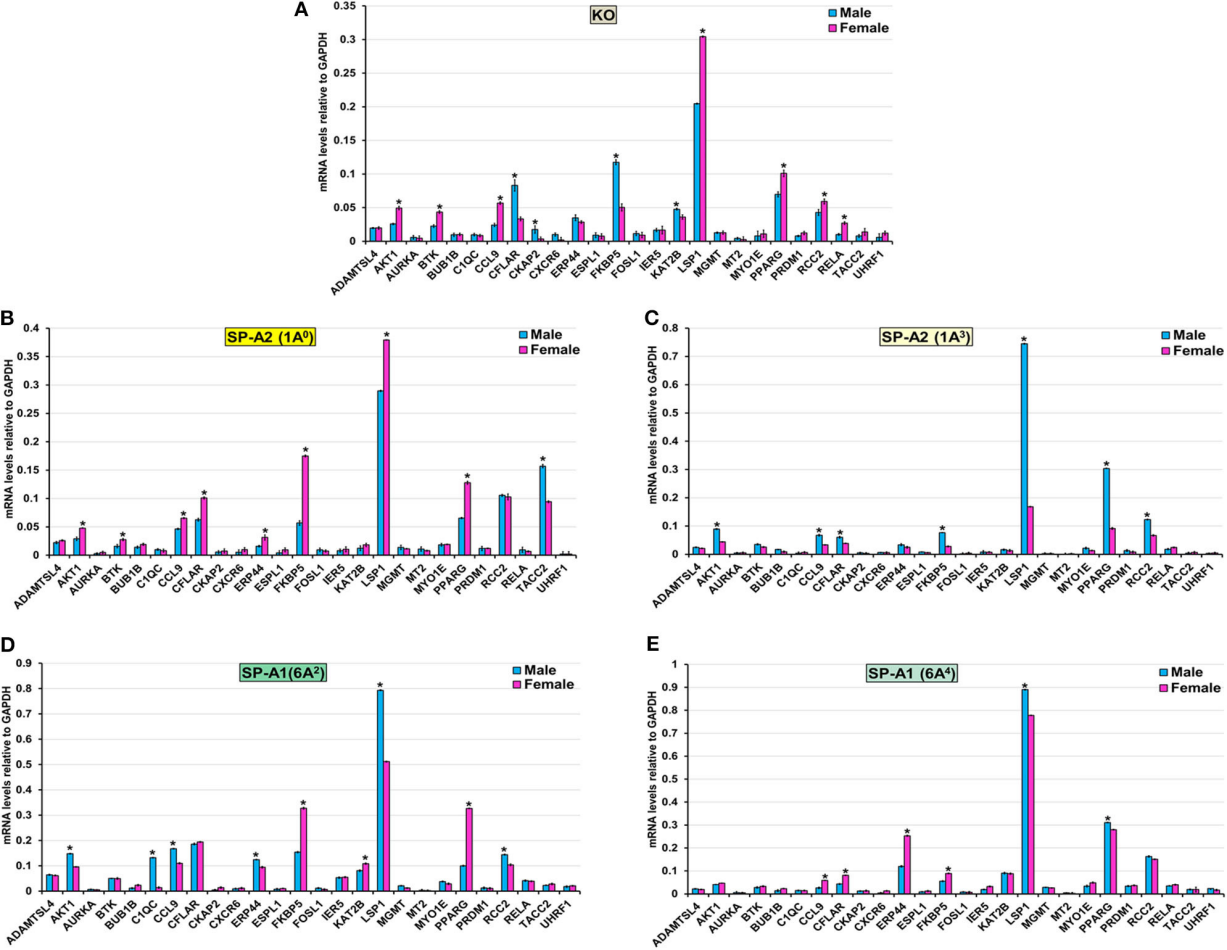
Effect of infection on the expression of genes involved in the TP53-node in SP-A1 (6A^2^, 6A^4^), SP-A2 (1A^0^, 1A^3^), and KO male and female. The expression level of genes involved in direct and indirect interactions with TP-53 shown in Figure 5A and Supplementary Figure 3 (ADAMTSL4, AKT1 AURKA, BTK, BUB1B, C1QC, CCL9, CFLAR, CKAP2, CXCR6, ERP44, ESPL1, FKBP5, FOSL1, IER5, KAT2B, LSP1, MGMT, MT2, MYO1E, PPARG, PRDM1, RCC2, RELA, TACC2, and UHRF1) were measured in KO **(A)**, SP-A2 (1A^0^) **(B)**, SP-A2 (1A^3^) **(C)**, SP-A1 (6A^2^) **(D)** and SP-A1 (6A^4^) **(E)**. The expression levels were normalized to GAPDH and the significant differences (*P* < 0.05) between sexes are noted with an asterisk (*).

The expression level of ADAMTSL4, AURKA, BUB1B, CXCR6, ESPL1, FOSL1, IER5, MGMT, MT2, MYO1E, PRDM1, and UHRF1 (KO, 1A^0^, 1A^3^, 6A^2^, and 6A^4^), TACC2 (KO, 1A^3^, 6A^2^, and 6A^4^), ERP44 (KO, 1A^3^), CKAP2 (1A^0^, 1A^3^, 6A^2^, and 6A^4^), KAT2B (1A^0^, 1A^3^, and 6A^4^), RCC2 (1A^0^, 6A^4^), BTK (1A^3^, 6A^2^, and 6A^4^), C1QC (KO, 1A^0^, 1A^3^, and 6A^4^), RELA (1A^0^, 1A^3^, 6A^2^, and 6A^4^), CFLAR (6A^2^), and AKT1(6A^4^) was similar between males and females in response to infection (Figure 7). However, in response to infection, male mice exhibited higher expression levels of CFLAR, and FKBP5 (KO, 1A^3^), AKT1, and CCL9 (1A^3^, 6A^2^), C1QC (6A^2^), LSP1 (1A^3^, 6A^2^, and 6A^4^), CKAP2, and KAT2B (KO), TACC2 (1A^0^), RCC2 (1A^3^, 6A^2^), PPARG (1A^3^, 6A^4^), and ERP44 (6A^2^) compared to females (Figure 7). The female mice exhibited higher expression levels of AKT1, BTK, CCL9, and LSP1 (KO, 1A^0^), PPARG (KO, 1A^0^, and 6A^2^), CFLAR, and ERP44 (1A^0^, 6A^4^), CCL9 (KO, 1A^0^, and 6A^4^), RCC2, and RELA (KO), KAT2B (6A^2^) and FKBP5 (1A^0^, 6A^2^, and 6A^4^) compared to males in response to infection (Figure 7).

#### Cell Cycle

**Figure 8:**
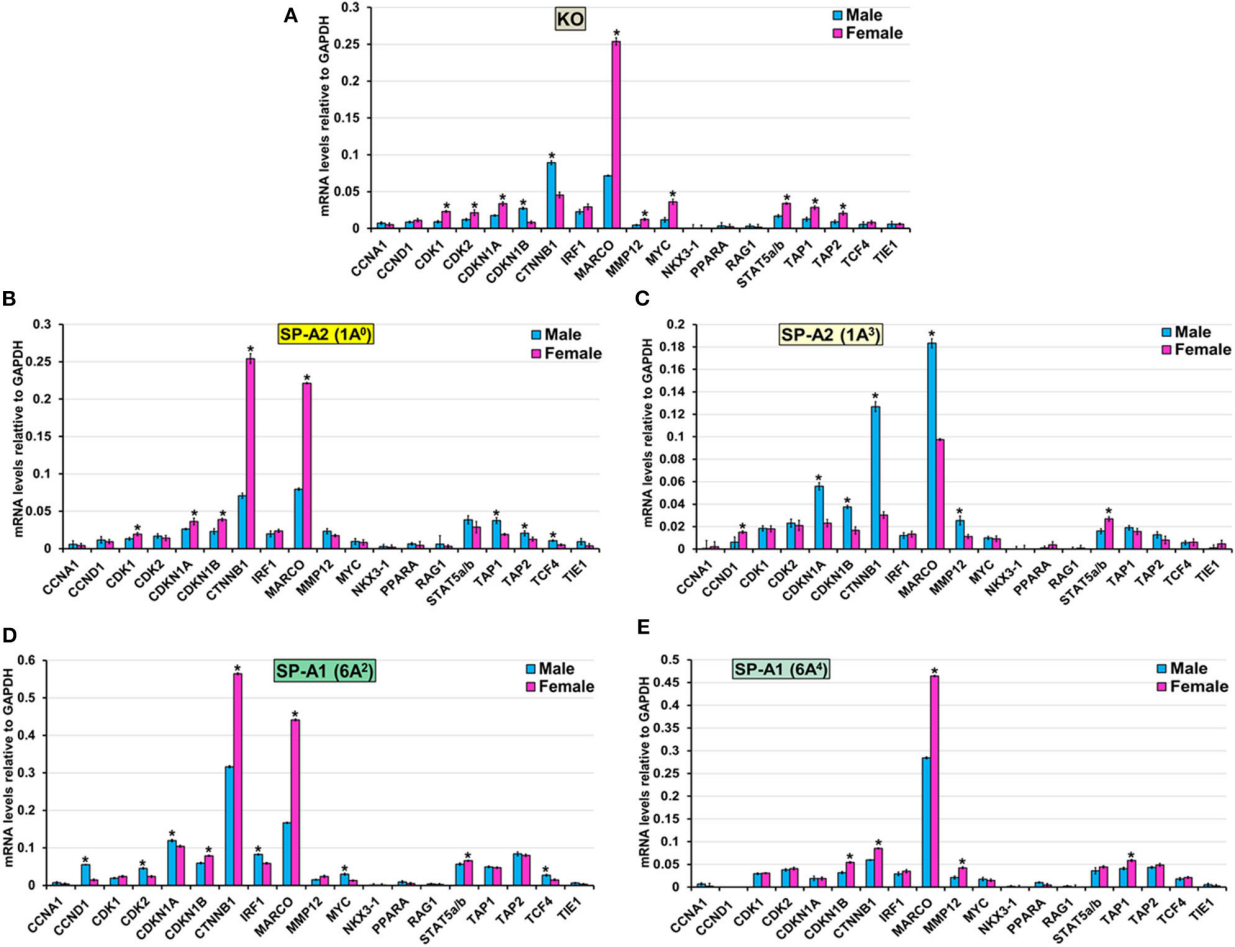
Effect of infection on the expression of genes involved in the Cell Cycle-node in SP-A1 (6A^2^, 6A^4^), SP-A2 (1A^0^, 1A^3^), and KO male and female. The expression level of genes involved in direct and indirect interactions with Cell Cycle shown in Figure 5A and Supplementary Figure 3 (CCNA1, CCND1, CDK1, CDK2, CDKN1A, CKDN1B, CTNNB1, IRF1, MARCO, MMP12, MYC, NKX3-1, PPARA, RAG1, STAT5a/b, TAP1, TAP2, TCF4, and TIE1) were measured in KO **(A)**, SP-A2 (1A^0^) **(B)**, SP-A2 (1A^3^) **(C)**, SP-A1 (6A^2^) **(D)** and SP-A1 (6A^4^) **(E)**. The expression levels were normalized to GAPDH and the significant differences (*P* < 0.05) between sexes are noted with an asterisk (*).

The expression level of CCNA1, NKX3-1, PPARA, RAG1, and TIE1 (KO, 1A^0^, 1A^3^, 6A^2^, and 6A^4^), CCND1, and IRF1 (KO, 1A^0^, 1A^3^, and 6A^4^) CDK2 (1A^0^, 1A^3^, and 6A^4^), CDK1 (6A^2^, 6A^4^, and 1A^3^), MMP12 (1A^0^, 6A^2^), MYC (1A^0^, 1A^3^, and 6A^4^), STAT5a/b (1A^0^, 6A^4^) TAP2 (6A^2^, 6A^4^, and 1A^3^), TAP1 (6A^2^, 1A^3^), CDKN1A (6A^4^) and TCF4 (KO, 1A^3^ and 6A^4^) was similar between males and females in response to infection (Figure 8). However, in response to infection, male mice exhibited higher expression levels of CDKN1B, and CTNNB1 (KO, 1A^3^), TCF4 (1A^0^, 6A^2^), TAP1, and TAP2, (1A^0^), CDKN1A, (1A^3^, 6A^2^), MARCO, and MMP12 (1A^3^), CCND1, CDK2, IRF and MYC (6A^2^) compared to females (Figure 8). The female mice exhibited higher expression levels of CDK1, and CDKN1A (KO, 1A^0^), CDK2 (KO), MARCO (KO, 1A^0^, 6A^2^, and 6A^4^), MMP12 (KO, 6A^4^), MYC (KO), STAT5a/b (KO, 1A^3^, and 6A^2^), TAP1 (KO, 6A^4^), TAP2 (KO), CDKN1B, and CTNNB1 (1A^0^, 6A^2^, and 6A^4^) and CCND1 (1A^3^) compared to males in response to infection (Figure 8).

In summary, the collective information of the significant changes in expression of genes in response to infection in AM cells from different SP-A variants and KO indicate sex-specific differences in expression of genes as well as, a differential impact of SP-A variants in the regulation of genes and may provide the foundation for the identification of sex-specific targets in response to infection.

## Discussion

Of the many types of macrophages found in the body, alveolar macrophages are in contact with external stimuli most frequently. As such, they are the primary effector cell for lung innate immunity and shown to be influenced by SP-A (29, 30, 35, 68–70), however, the extent of this effect is not fully understood. Furthermore, sex-dependent survival in wild type and SP-A-KO mice in response to *K. pneumoniae* infection (29, 30) as well as sex differences in survival in mice carrying different human SP-A1 and SP-A2 variants (32) were observed. In this study, we wished to investigate AM gene expression and sex differences in hTG mice, SP-A1, SP-A2, and SP-A-KO in response to *K. pneumoniae*. We found (a) significant differences in gene expression of SP-A2 (1A^0^) AM at 6, 18, and 24 h post-infection, as well as sex differences at 6 and 24 h post-infection, but not at 18 h. (b) significant sex differences in AM gene expression of SP-A2 (1A^3^), SP-A2 (6A^2^, 6A^4^), and SP-A-KO mice at 6 h post-infection; (c) of the three pathways (TNF, TP-53, and cell cycle signaling nodes) studied here, all variants except SP-A2 (1A^3^) female, showed significance for at least 2 of these pathways, and KO male showed significance for all three pathways; (d) though the expression profile of validated genes was variant-specific, a similarity in the gene expression profile of KO and SP-A1 mice was observed.

In the present study, we build and extend on previous findings where functional differences of human SP-A1 and SP-A2 variants were investigated, in terms of their differential impact on AM miRNome after oxidative stress (64), the AM proteomic profile (61), and post-infection survival (32). The gene expression differences observed at 6 h post-infection are reminiscent with AM proteomics findings where mice carrying single gene variants of SP-A1 and SP-A2 exhibited variant-specific protein expression profile (61). More similarities in gene expression were observed in KO and SP-A1 compared to SP-A2. KO and SP-A1 have been shown previously to be more similar in regards to proteomic profile, miRNome of AM, and airway function but distinct from SP-A2 in response to infection with or without oxidative stress (62, 64, 73). IPA analysis of genes involved in molecular and cellular functions revealed differential expression of genes related to cell morphology, cell function and maintenance, cell development, and movement in both males and females. Out of all the nodes that appeared in IPA, we chose three nodes: TNF, TP53, and cell cycle signaling, for further study. These contained direct interactions for 4 or more molecules in at least one of the SP-A variants of either male or female. The TNF node for SP-A2 (1A^0^) males, although did not fulfill the selection criteria of direct interactions, was included for further study because of the large number of molecules (40–70% more compared to other variants) showing indirect interactions. Subsequently, genes that had direct or indirect interactions within these pathways were validated and discussed below.

### Sex Differences in Gene Expression

#### TNF Node: Pro-Inflammatory Responses

After infection, the 1A^3^, 6A^2^ variants, and KO male mice, and female 1A^0^ and 6A^4^ variants (but not males) exhibited higher expression levels of CXCL2 in response to infection. CXCL2 is an antimicrobial cell-signaling cytokine and a chemoattractant with a pro-inflammatory function and linked to ventilator (80) and hyperoxia-induced acute lung injury (81), and it contributes to chemotaxis, and immune and inflammatory response after infection (82). Hyperoxia increases neutrophil recruitment and lung injury that parallels the expression of CXCL2. Similarly, a higher number of neutrophils was observed in BAL of KO males in response to infection and ozone-induced oxidative stress (30). Moreover, inhibition of CXCL2 receptors has been shown to attenuate hyperoxia-induced inflammation and improved survival (81). Whether the higher expression of CXCL2 in the present study along with high neutrophil in the BAL of KO male observed in the previous study (30, 31) explain the lowest survival for KO male remains to be determined. Of interest, 1A^3^, and 6A^2^ males with high CXCL2 also showed low survival post-infection (32). However, in the present study, we observed high expression of CXCL2 in 1A^0^ and 6A^4^ female mice but these mice have shown to have the highest and lowest survival rate, respectively, post-infection (32). Although neutrophil levels were not assessed in that study, this observation points to potential mechanistic complexities incurred by SP-A genetics. Moreover, the CXCL2 gene expression in the present study varied as a function of sex and variant, and further study is needed to elucidate the complexity of the underlying mechanisms.

Male SP-A2 (1A^0^, 1A^3^) variants, and female KO and SP-A1 variant (6A^2^, 6A^4^) mice showed increased expression of SAMHD1 gene. An upregulation of SAMHD1 by LPS induced acute lung injury in as early as 6 h of post-stimulation and was thought to be one of the early cellular responses and an effector of innate immunity after infection (83). This particular gene plays an important role in the regulation of innate immune response via type 1 interferons (84). These interferons also activate JAK/STAT signaling pathways. Similar to the SAMHD1 gene, we observed higher expression of STAT1 and STAT3 genes in male SP-A2 (1A^0^, 1A^3^) and female KO, and SP-A1 (6A^2^, 6A^4^) mice. The STAT genes are a family of cytoplasmic transcription factors that are activated by various cytokines, growth factors, and other stimuli and phosphorylated by many protein kinases (85). Interferon-mediated activation of STAT3 in macrophages is an indispensable mechanism to prevent inflammation in mice (85). Moreover, increased STAT3 mRNA level at 4 h and 18 h, as well as increased IL-6 (a STAT3 regulator) at 18 h, in AM of SP-A2 male (but not female) mice after oxidative stress was observed (64) indicating a time, sex, and SP-A variant dependent changes in AM. We postulate that microbial infection, as in the present study, induces Janus kinase (JAK) family that mediates phosphorylation of STAT1 and STAT3. These, in turn, translocate into the nucleus, resulting in increased expression of IFN-stimulated genes, such as SAMHD1, that modulate host immune responses (86). Taken together, these indicate the potential role of SP-A genetics mediating AM gene expression in response to infection. Although the underlying mechanisms remain unclear, it is likely to be an interplay of sex and SP-A genetic variants.

The ingenuity pathway analysis revealed TNF node networks for SP-A2 (1A^0^) and KO males only. Although the network pathway is very complex there are a couple of interesting features. a) In KO male, the NF-κB complex is shown to be activated directly by the IKK complex 6 h post-infection (Supplementary Figure 3). It has been shown that in the classic NF-κB pathway, IKK molecules get phosphorylated in response to various stimuli such as bacterial or viral products, inflammatory cytokines, and oxidative stress (87). This modification allows their polyubiquitination and destruction by the proteasome. As a consequence, free NF-κB enters the nucleus and activates the transcription of genes that participate in the immune and inflammatory response after infection (87). In contrast, in SP-A2 mice, the NFκB complex does not appear in the TNF node pathway and the IKK complex appears to be directly activated by BCL10, the expression of which was similar in males and females in SP-A2 and KO male 6 h post-infection (Figure 6). The BCL10 is an upstream activator of the IKK complex in the innate immunity pathways, which in turn activates the NF-κB complex (88). SP-A is shown to activate the NF-κB complex (89). Moreover, a recent study of BAL proteomic profile after infection and oxidative stress showed upregulation of proteins that regulate the NF-κB pathway in SP-A1 and SP-A2 males but not in females (90). Of interest, ozone (O_3_)-induced oxidation of SP-A decreases its ability to activate the classic NF-κB pathway (91) and O_3_ exposure of THP-1 cells resulted in a decrease in SP-A mediated THP-1cell responsiveness, indicating an alternative mechanisms are involved when both THP-1 cells (a macrophage-like cell line) and SP-A are exposed to ozone simultaneously (91). We postulate that oxidation of SP-A, particularly of SP-A2, is responsible for the activation of a non-classic NF-κB pathway in SP-A2 (1A^0^) as shown in IPA (Figure 5). Of interest, a previous study of ozone-induced oxidative injury showed 18 h after ozone exposure a downregulation of AM miRNAs that target NF-κB in SP-A2 (1A^0^) male but not in KO male (64), indicating a potential upregulation of NF-κB in SP-A2 (1A^0^). However, the mRNA level of IKKβ decreased and NF-κB remained unchanged at 18 h post ozone exposure. Taken together, these observations indicate that activation of IKKB-NF-κB pathway is delayed in response to pulmonary insults in SP-A2 (1A^0^) male compared to KO male, whether this is due to SP-A2 oxidation (48, 92) remains to be determined. b) In SP-A2 (1A^0^) male, interleukin-1 (IL-1) and IL1 receptor antagonist (IL1RN) get indirectly activated at 6 h post-infection (Figure 5). SP-A induces IL-1 production in THP cell line after a short term exposure (2 h) of LPS (93). SP-A upregulates the IL-1 receptor-associated kinase M in response to long term exposure (6–24 h) of LPS which in turn suppresses the production of cytokines in alveolar macrophages (94, 95). The upregulation of IL-1 receptor-associated kinase M starts after 6 h of exposure to SP-A and gets maximized at 12 h. In the present study at 6 h post-infection, IPA showed indirect activation of both IL1 and IL1RN in AM of SP-A2 (1A^0^) male. Considering the role of SP-A in both pro- and anti-inflammation, we speculate that the balance starts to tilt toward anti-inflammatory response in AM at 6 h post-infection.

#### TP-53-Node

Although the TP-53 node is shown to associate with lung cancer (96), we observed in the present study a significant contribution of the TP-53 node by IPA analysis which is based on existing information about gene functions. Previous studies have shown that SP-A1 (6A^4^) and SP-A2 variants are associated with lung carcinoma (97–99). Therefore, it is possible that the TP-53 node genes are also involved and upregulated after infection. We found collectively among SP-A variants several genes such as AKT1, BTK, CCL9, PPARG, and RELA to exhibit higher expression in females, whereas, CFLAR, C1QC, LSP1, CKAP2, KAT2B, TACC2, and RCC2 exhibited higher expression in males after infection (Figure 7). These genes may represent mechanisms yet to be understood and new knowledge of processes operating during the early phase of infection. After qRT-PCR validation, we found that KO and 1A^3^ males, and 1A^0^, 6A^2^, and 6A^4^ females exhibited a higher expression of the FKBP5 gene. The FKBP5 gene encodes an immunophilin protein that plays a role in immunoregulation and basic cellular processes involving protein folding and trafficking. Furthermore, the FKBP5 gene is related to glucocorticoid receptor regulatory network and PI3K/Akt signaling pathways. A recent study showed an association of the FKBP5 gene with chronic obstructive pulmonary disease (COPD), and those with a particular genetic variant respond better to inhaled corticosteroids (100). However, the influence of sex was not studied. COPD is an inflammatory, non-reversible obstructive lung disorder with abnormal lung function showing obstruction to airflow and increased resistance (101). The gram-negative bacterial infection, particularly K. pneumoniae, is a common cause of acute exacerbation of COPD, and baseline pulmonary function is the strongest predictor of the outcome of acute exacerbation (102, 103). Of note, recently we showed a significant increase in resistance and decrease in lung compliance (the ease with which lung can be stretched) at 18 h after K. pneumoniae infection in females compared to males in KO, SP-A1 (6A^2^, 6A^4^), and SP-A2 (1A^0^, 1A^3^) variants mice (73). However, the pattern changed with males showing increased resistance in response to methacholine challenge after infection, especially for single SP-A1 or SP-A2 variants (73). Male sex is associated with severe COPD, and association of severe COPD and SP-A1 variant has been shown in smokers (104). The differential impact of SP-A genetic variants and sex on expression of the FKBP5 gene in the present study and on lung function in previous study (73), along with human studies shows predisposition of male sex and the FKBP5 gene for severe COPD, it will be interesting to explore further i.e., study the role of SP-A-mediated change in the FKBP5 gene in COPD patients, particularly in males.

Considering the diverse functional implications of SP-A in various lung diseases, it is not surprising that SP-A is also shown to regulate tumor microenvironment by inducing the production of inflammatory cytokines and controlling the polarization of tumor-associated macrophages in lung cancer (105). In the current study, we did not observe any changes in the expression levels of genes involved in the regulation of the mitotic and meiotic cell division, differentiation, and growth (AURKA, BUB1B, ESPL1, FOSL1, IER5, and MGMT). However, expression levels of genes involved in AM mediated inflammation and cytokine production (BTK, C1QC, CCL9, FKBP5, LSP1, PRDM1, and PPARG) were significantly increased. Although the higher expression of genes that regulate inflammation and cytokine production in the present study are due to bacterial infection rather than any true carcinogenic modulation, deranged immunity to foreign (i.e., bacterial, virus, toxic inhalants) or endogenous tumor-associated antigens is responsible for at least 15% of lung cancer (106). Based on the available information, we postulate that SP-A contributes to the tumor microenvironment by controlling AM genes that regulate inflammation and cytokine production.

#### Cell Cycle

Bacterial pathogens have shown to employ a variety of strategies to manipulate the host cell cycle (107). In the present study, the majority of the genes (CCNA1, MYC, NKX3-1, PPARA, TCF4, and TIE1) that regulate various phases of cell division are unchanged, except for the high expression of CDK1 and CDK2 genes in SP-A2 (1A^0^) female and SP-A1 (6A^2^) male, respectively. Of note, previous studies of oxidative stress-mediated change in AM miRNome showed downregulation of miRNAs that upregulate the CDK2 mRNA and other cell cycle genes in co-ex males (mice that expressed both SP-A1 and SP-A2) but not in a single SP-A variant mice indicating the need for both SP-A1 and SP-A2 to regulate cell cycle function in response to oxidative stress (64, 76). In response to infection, both gene products are needed to regulate cell cycle genes as shown for the AM miRNome in response to oxidative stress (76).

The majority of genes (CTNNB1, MARCO, STAT5a/b, TAP1, and TAP2) that regulate proliferation and differentiation of immune cells showed a differential higher expression in KO and SP-A variants in response to infection. Specifically, the MARCO gene exhibited higher expression in 1A^3^ male and KO, 1A^0^, 6A^2^, and 6A^4^ female. MARCO (Macrophage receptor with collagenous structure) is a distinct member of the scavenger receptor family and plays an important role in innate immune defense by acting as pattern recognition receptors (108). Though it is expressed only in some subpopulations of macrophage, it is shown to be upregulated in AM and correlates with survival after *Streptococcus pneumoniae* lung infection (109). Moreover, the downregulation of MARCO in AM is associated with a decreased clearance and increased susceptibility of *Streptococcus pneumoniae* after influenza infection (110). In the present study, MARCO is upregulated in AM of female mice except for 1A^3^. Previously, a better survival for female compared to male after *K. pneumoniae* infection was observed (32). We speculate that the upregulation of MARCO contributes to better survival for females. Of interest, the upregulation of MARCO in 1A^3^ male and KO showing lower survival is intriguing and puzzling, and further study is needed to explore the role of MARCO in *K. pneumoniae* infection. Moreover, we observed upregulation of the transporter associated with antigen processing 1 (TAP1) and TAP2 genes in 1A^0^ male and KO female. These proteins are located on the endoplasmic reticulum (ER) membrane and are necessary to translocate viral and bacterial peptides from the cytosol to the ER and ensure proper loading of those peptides to major histocompatibility complex (MHC) class I that gets presented to cytotoxic T cells (111). The observation of chronic bacterial lung infections in TAP1 and TAP2 deficient patients indicates that presentation of bacterial antigens by MHC on AM is defective (112), leading to ineffective clearance of bacteria from the lung. Furthermore, an increase in TAP gene expression and TAP activity in response to infection has been observed (113). Currently, it is unclear why the upregulation of TAP genes are specific to 1A^0^ male and KO female, particularly in light of the survival study that showed the highest survival for 1A^0^ female and the lowest survival for KO male (32). We speculate that if a more vigorous response occurs in mice of a given sex and/or mice that carry a specific SP-A variant (or lack SP-A as is the case in KO) making them presumably less capable to effectively handle or control the infection, then the survival of these mice is negatively affected.

Moreover, other genes such as CCNA1, CCND1, IRF1, NKX3-1, PPARA, RAG1, TIE1, CDK1, MMP12, MYC, STAT5a/b, CDKN1A and TCF4 that are part of the cell cycle node had sex and variant-specific differential gene expression changes in response to infection. Previous studies of AM gene profiling after infection (114, 115) have shown genes that regulate cell cycle to differ from the genes identified here. This is likely due to different conditions, type, and timing of infection.

Our study has few limitations: (a) we validated genes identified by IPA that had direct interactions in the TP53, TNF, and cell cycle signaling nodes, but we did not measure the protein levels. However, there is a significantly higher correlation between mRNA and protein level for genes that are differentially expressed (116), and we speculate that the mRNA levels of the differentially expressed genes studied here correlate in most (if not all) cases with protein levels, (b) the molecular mechanisms of the identified pathways were not studied, and (c) though the level of SP-A1 and SP-A2 is similar in single gene variants (Figure 1), we did not study the impact of varying amount of SP-A1 and SP-A2 on AM gene expression in response to infection. Previously, it has been shown, that the ratio of SP-A1 to total SP-A differs based on lung pathology, age, and sex (117, 118), and this may have consequences on AM given the relative functional differences of SP-A1 and SP-A2. Nonetheless, the results of the present study further shed light on the complexities of SP-A genetic variants on AM expression as well as the role of sex on AM after infection. Differences also exist in the receptors sensing infections.

#### Pros and Cons of Human Transgenic Mouse Model

There are significant differences between mice and humans that include aspects of the immune system development, activation, and response to infection (10, 119, 120). Differences also exist in the receptors sensing infections (121), and the ligand specificities and affinities of TLRs (122, 123). However, several studies have shown that the mouse pneumonia model recapitulates key features of Klebsiella-induced pneumonia in humans as well as differences. Mice have shown increased neutrophils in BAL in response to *K. pneumoniae* infection (124) similar to that of human BAL (125). It has been observed that although the phagocytic activity level of the rodent (rat) and human alveolar macrophages differs, the phagocytic activity of both rat and human AMs was enhanced in the presence of human SP-A variants, with SP-A2 exhibiting higher activity than SP-A1 (48). Similar observations have been made with mouse AMs (our unpublished preliminary data). Furthermore, similarities have been observed in survival in humans and mice with regards to SP-A variants. A better survival in the first year after lung transplantation was observed in humans if the transplanted lung carried a specific SP-A2 (1A^0^) variant (126) and this variant was also found to associate with better survival in mice after infection (32). This further supports the usefulness of the SP-A1 and SP-A2 transgenic mice to gain further insight into the human conditions.

#### Overall Comments

SP-A1 and SP-A2 exhibit gene-specific amino acid differences located within the collagen-like region at positions 66, 73, 81, and 85. The presence of cysteine at 85 position of SP-A1 and arginine for SP-A2 has a remarkable impact on SP-A structure and function (51). SP-A2 exhibits a significant higher activity of host-defense functions, such as phagocytosis (47, 48, 59) as well as in survival in response to infection (32) compared to SP-A1, whereas SP-A1 exhibits higher efficiency in pulmonary surfactant structural reorganization and in the inhibition of surfactant function by serum proteins (66). Moreover, both SP-A1 and SP-A2 are required to make tubular myelin, an extracellular structural form of surfactant (52). Studies of the AM miRNome in response ozone-induced oxidative stress showed that although SP-A1, by itself did not have any major effect on the AM miRNome under the studied conditions (64), in the presence of SP-A2, modulated gene expression in certain pathways (76). For the most part SP-A1 and SP-A2 exhibit similar functions but their activity level differs significantly. Although in human both genes are expected to be expressed, the relative levels of each may differ significantly (117), and these differences have been correlated with various lung disease such as asthma (118) and cystic fibrosis as well as culture positive bronchoalveolar lavage samples (117). The corollary to this that as the individual levels may vary, the SP-A1 and SP-A2 function in the lung may also vary especially in response to pressures and thus modify disease susceptibility of the host.

A recent study showed that SP-A1 and SP-A2 differentially bind to AM. The maximal binding (Bmax) of SP-A2 to AM was shown to be 2-3 times higher than that of SP-A1 binding (127). Moreover, SP-A2 exhibits a higher ability to bind phagocytic cells, such as AMs and THP-1 cells than SP-A1, but neither SP-A1 or SP-A2 bound CHO cells, a non-phagocytic cell line (32), further supporting a differential cell-specific receptor binding. Although the mechanistic details of how the differential binding may differentially affect AM function have not been investigated, several studies have shown that SP-A1 and SP-A2 exhibit differences in the phagocytic index of AM in *ex-vivo* studies with SP-A2 exhibiting higher activity (47, 48, 59).

In the present study, although in general, the gene expression profile and pathway analysis is distinct for each variant, we observed a few interesting patterns: (1) the expression profile of the genes that are involved in TNF node pro-inflammatory pathway is more similar for KO and SP-A1 than KO and SP-A2, particularly for females with 40% of the validated genes showing significantly increased expression (Figure 6). A similarity in the AM proteomic profile of SP-A1 (6A^2^) and KO has been previously observed, and the AM proteomic profile of SP-A2 (1A^0^) was similar to that of WT in basal conditions (61, 62). Functionally, SP-A2 enhances bacterial cell association, phagocytosis, and cytokine production by AM more effectively than SP-A1 (47, 48, 57), and exhibits a significantly better survival rate after infection (32). Residue 85 in SP-A1 (cysteine) and in SP-A2 (arginine) plays an important role in phagocytosis and other SP-A-mediated functions (51). The cysteine may further lead to structural instability in SP-A1 (128). Thus, the fact that the gene expression profile of the pro-inflammatory pathway is similar in KO and SP-A1 variants may not be that surprising. Though not studied here, we speculate that compared to SP-A1 and KO, the gene expression profile of the pro-inflammatory pathway would be more similar in WT and SP-A2 variants. (2) The expression profile of genes that are involved in TP53 node are similar in KO and SP-A2 (1A^0^) (Figure 7). Considering previous observations of similarity between KO and SP-A1 variants, the finding of similarity in TP53 node of KO and SP-A2 (1A^0^) is surprising and intriguing. (3) No significant differences in the expression pattern of genes that are involved in the cell cycle signaling node were observed. (4) Although canonical pathways appear distinct for each variant, the pattern is similar for KO and SP-A1 (6A^2^) variant with the SP-A1 (6A^4^) showing only the TP53 node to be significant. Whereas, for the SP-A2 variants, the picture was quite different, with the SP-A2 (1A^3^) showing only the cell cycle signaling node for females and none for males (Supplementary Figure 3).

In summary, although, collectively, the total amino acid differences among human SP-A1 and SP-A2 genetic variants is small (*n* = 10 with only four of them being gene-specific), their functional impact on AMs is varied and extensive and includes changes in the function, actin cytoskeleton, proteome, miRNome, and the gene expression profile and pathways involved (present study). Humanized transgenic mice, each carrying a different SP-A1 or SP-A2 variant have been shown to differentially affect lung function and survival after infection in a sex-dependent manner. These observations together beg the consideration of SP-A genotype in human lung diseases where dysregulation of inflammatory process and host defense, in general, are part of the underlying causes. In fact, a given SP-A2 genotype/variant, shown previously to associate with better mouse survival after infection (32), is also associated with better survival in lung transplant patients especially in the first year after lung transplant, which is the most critical time perhaps due to dysregulation of inflammation and host defense (126).

## Data Availability Statement

The datasets generated for this study are included in the article and the Supplementary Material, and have been deposited in the Gene Expression Omnibus repository GSE144595 (https://www.ncbi.nlm.nih.gov/geo/query/acc.cgi?&acc=GSE144595).

## Ethics Statement

The protocol used in this study was evaluated and approved by the Pennsylvania State University College of Medicine Institutional Animal Care and Use Committee and confirmed to the guidelines of the National Institute of Health on the Care and Use of Laboratory Animals.

## Author Contributions

NT performed experiments, mouse line maintenance, breeding, and infection, run statistics, analyzed and synthesized the data, and contributed to the manuscript writing. YK performed RNA sequence analysis. CG contributed to manuscript writing. JF designed the study and provided oversight to the entire project, involved in data analysis, integration, and writing of the manuscript. All authors read and approved the final manuscript.

## Conflict of Interest

The authors declare that the research was conducted in the absence of any commercial or financial relationships that could be construed as a potential conflict of interest.

## Supplementary Material

Supplementary Figure 1 Venn diagrams show the number of genes identified in comparisons of combined males and females from mice each carrying a different human variant in response to *K. pneumoniae*. **(A)** Comparison between SP-A1 (6A^2^ vs. 6A^4^). Out of 196 and 494 genes identified from 6A^2^ and 6A^4^ respectively, 5 are identified to be in common in both the SP-A1 gene-specific variants, 191 are specific to 6A^2^, and 489 are to 6A^4^. **(B)** Comparison between SP-A2 (1A^0^ vs. 1A^3^). Out of 276 and 397 genes identified from 1A^0^ and 1A^3^, respectively, 31 are identified to be in common in both the SP-A2 gene-specific variants, 245 are specific to 1A^0^ and 366 are to 1A^3^. **(C)** Comparison of genes between SP-A2 (1A^0^) vs. SP-A1 (6A^2^). Out of 276 and 196 genes identified from 1A^0^ and 6A^2^, respectively, 10 are identified to be in common in the SP-A1 (6A^2^) and SP-A2 (1A^0^) gene variants, 266 are specific to 1A^0^ and 186 are to 6A^2^. **(D)** Comparison of genes between SP-A2 (1A^0^) vs. SP-A1 (6A^4^). Out of 276 and 494 genes identified from 1A^0^ and 6A^4^, respectively, 15 are identified to be in common in the SP-A1 (6A^4^) and SP-A2 (1A^0^) gene variants, 261 are specific to 1A^0^ and 479 are to 6A^4^. **(E)** Comparison of genes between SP-A2 (1A^3^) vs. SP-A1 (6A^2^). Out of 397 and 196 genes identified from 1A^3^ and 6A^2^, respectively, 14 are identified to be in common in the SP-A1 (6A^2^) and SP-A2 (1A^3^) gene variants, 384 are specific to 1A^3^ and 182 are to 6A^2^. **(F)** Comparison of genes between SP-A2 (1A^3^) vs. SP-A1 (6A^4^). Out of 397 and 494 genes identified from 1A^3^ and 6A^4^, respectively, 31 are identified to be in common in the SP-A1 (6A^4^) and SP-A2 (1A^3^) gene variants, 366 are specific to 1A^3^ and 463 are to 6A^4^.

Supplementary Figure 2 Venn diagram comparisons of the number of genes from different variants vs. KO. **(A)** Comparison between SP-A2 (1A^0^) vs. KO. Out of 276 and 858 genes identified from 1A^0^ and KO, respectively, 40 are identified to be in common in the SP-A2 (1A^0^) and KO, 236 are specific to 1A^0^ and 818 are to KO. **(B)** Comparison between SP-A2 (1A^3^) vs. KO. Out of 397 and 858 genes identified from 1A^3^ and KO, respectively, 142 are identified to be in common in the SP-A2 (1A^3^) and KO, 255 are specific to 1A^3^ and 716 are to KO. **(C)** Comparison between SP-A1 (6A^2^) vs. KO. Out of 196 and 858 genes identified from 6A^2^ and KO, respectively, 27 are identified to be in common in the SP-A1 (6A^2^) and KO, 169 are specific to 6A^2^ and 831 are to KO. **(D)** Comparison between SP-A1 (6A^4^) vs. KO. Out of 494 and 858 genes identified from 6A^4^ and KO, respectively, 42 are identified to be in common in the SP-A1 (6A^4^) and KO, 452 are specific to 6A^4^ and 816 are to KO.

Supplementary Figure 3 Ingenuity Pathway Analysis for gene expression in males and females exposed to *K. pneumoniae* infection for 6 h in SP-A1 (6A^2^, 6A^4^), SP-A2 (1A^3^) and KO. Diagrams of biological networks of selected genes whose expression was ≥2-fold in the AM of male and female mice after infection are shown in color. *Left*, genes and pathways in male mice; *right*, genes and pathways in female mice. The diagrams show reported direct (solid lines) or indirect (dashed lines) gene interactions. Each gene or group of genes is represented as a node. Molecules that are significantly altered ≥2 are represented as node in red. Node shapes represent functional classes of gene products: Square for cytokines, Concentric (double) circle for complex/group, Diamonds for enzymes and peptidases, Ovals for transcription regulators and Transmembrane receptors, Triangle for phosphatases and Kinases, Rectangles for ligand-dependent nuclear receptors, G-protein coupled receptors, and ion channels, Trapezoids for transporters and microRNAs.

Supplementary File 1 Identification of genes from AMs of SP-A2 (1A^0^) males and females in response to *K. pneumoniae* infection at different time points (6, 18, and 24 h).

Supplementary File 2 Identification of genes from AMs of SP-A1 (6A^2^), SP-A1 (6A^4^), SP-A2 (1A^3^), and KO males and females in response to *K. pneumoniae* infection after 6 h.

Supplementary File 3 Comparison of genes from AMs of SP-A2 (1A^0^), SP-A2 (1A^3^), SP-A1 (6A^2^), SP-A1 (6A^4^), and KO males and females in response to *K. pneumoniae* infection after 6 h.

## Abbreviations

ADAMTSL4: ADAMTS-like 4
AKT1: AKT serine/threonine kinase 1
AM: Alveolar macrophages
ANOVA: Analysis of variance
AURKA: aurora kinase A
BAL: bronchoalveolar lavage
BCL3: B cell leukemia/lymphoma 3
BCL10: B cell leukemia/lymphoma 10
BTK: Bruton agammaglobulinemia tyrosine kinase
BUB1B: mitotic checkpoint serine/threonine kinase
C1QC: complement component 1, q subcomponent, C chain
CCL9: chemokine (C-C motif) ligand 9
CCNA1: cyclin A1
CCND1: cyclin D1
CCRL2: chemokine (C-C motif) receptor-like 2
CDK1: cyclin-dependent kinase 1
CDK2: cyclin-dependent kinase 2
CDKN1A: cyclin dependent kinase inhibitor 1A
CDKN1B: cyclin dependent kinase inhibitor 1B
CFLAR: CASP8 and FADD like apoptosis regulator
CKAP2: cytoskeleton associated protein 2
CTTNB1: catenin alpha 1
CXCL2: chemokine (C-X-C motif) ligand 2
CXCR6: chemokine (C-X-C motif) receptor 6
ERP44: endoplasmic reticulum protein 44
ESPL1: extra spindle pole bodies 1
FKBP5: FK506 binding protein 5
FOSL1: fos-like antigen 1
GBP2: guanylate binding protein 2
IER5: immediate early response 5
IFITM2: interferon induced transmembrane protein 2
IRF1: interferon regulatory factor 1
IPA: Ingenuity Pathway Analysis
KAT2B: K(lysine) acetyltransferase 2B
KO: knock-out
LSP1: lymphocyte specific 1
MARCO: macrophage receptor with collagenous structure
MGMT: O-6-methylguanine-DNA methyltransferase
MMP12: matrix metallopeptidase 12
MT2: metallothionein 2
MYC: MYC proto-oncogene
MYD88: myeloid differentiation primary response gene 88
MYO1E: myosin IE
NF-kB: Nuclear factor kappa-light-chain-enhancer of activated B cells
NKX3-1: NK3 homeobox 1
PPARA: peroxisome proliferator activated receptor alpha
PPARG: peroxisome proliferator activated receptor gamma
PRDM1: PR domain containing 1, with ZNF domain
PSMB8: proteasome (prosome, macropain) subunit, beta type 8
RAG1: recombination activating 1
RCC2: regulator of chromosome condensation 2
RELA: RELA proto-oncogene, NF-kB subunit
RSAD2: radical S-adenosyl methionine domain containing 2
RTP4: receptor transporter protein 4
SAMHD1: SAM domain and HD domain, 1
*SFTPA1*: gene encoding SP-A1
*SFTPA2*: gene encoding SP-A2
SP-A: surfactant protein A
STAT1: signal transducer and activator of transcription 1
STAT3: signal transducer and activator of transcription 3
STAT5a/b: signal transducer and activator of transcription 5A/B
TACC2: transforming, acidic coiled-coil containing protein 2
TAP1: transporter 1, ATP-binding cassette, sub-family B
TAP2: transporter 2, ATP-binding cassette, sub-family B
TCF4: transcription factor 4
TIE1: tyrosine kinase with immunoglobulin-like and EGF-like domains 1
UHRF1: ubiquitin-like, containing PHD and RING finger domains 1
ZFP36L1: zinc finger protein 36, C3H type-like 1.

## References

[1] Podschun R Ullmann U. *Klebsiella* spp. as nosocomial pathogens: epidemiology, taxonomy, typing methods, and pathogenicity factors. Clin Microbiol Rev. (1998) 11:589–603. 9767057 10.1128/cmr.11.4.589 PMC88898

[2] Kofteridis DP Papadakis JA Bouros D Nikolaides P Kioumis G Levidiotou S. Nosocomial lower respiratory tract infections: prevalence and risk factors in 14 Greek hospitals. Eur J Clin Microbiol Infect Dis. (2004) 23:888–91. 10.1007/s10096-004-1245-y 15558346

[3] Bagley ST. Habitat association of Klebsiella species. Infect Control. (1985) 6:52–8. 10.1017/s0195941700062603 3882590

[4] Rock C Thom KA Masnick M Johnson JK Harris AD Morgan DJ. Frequency of *Klebsiella pneumoniae* carbapenemase (KPC)-producing and non-KPC-producing Klebsiella species contamination of healthcare workers and the environment. Infect Control Hosp Epidemiol. (2014) 35:426–9. 10.1086/675598 24602950 PMC4030386

[5] Dao TT Liebenthal D Tran TK Ngoc Thi Vu B Ngoc Thi Nguyen D Thi Tran HK. Klebsiella pneumoniae oropharyngeal carriage in rural and urban Vietnam and the effect of alcohol consumption. PLoS ONE. (2014) 9:e91999. 10.1371/journal.pone.0091999 24667800 PMC3965401

[6] Paczosa MK Mecsas J. *Klebsiella pneumoniae*: going on the offense with a strong defense. Microbiol Mol Biol Rev. (2016) 80:629–61. 10.1128/mmbr.00078-15 27307579 PMC4981674

[7] Munoz-Price LS Poirel L Bonomo RA Schwaber MJ Daikos GL Cormican M. Clinical epidemiology of the global expansion of *Klebsiella pneumoniae* carbapenemases. Lancet Infect Dis. (2013) 13:785–96. 10.1016/s1473-3099(13)70190-7 23969216 PMC4673667

[8] Mizgerd JP. Molecular mechanisms of neutrophil recruitment elicited by bacteria in the lungs. Semin Immunol. (2002) 14:123–32. 10.1006/smim.2001.0349 11978084

[9] Burns AR Smith CW Walker DC. Unique structural features that influence neutrophil emigration into the lung. Physiol Rev. (2003) 83:309–36. 10.1152/physrev.00023.2002 12663861

[10] Mizgerd JP. Acute lower respiratory tract infection. N Engl J Med. (2008) 358:716–27. 10.1056/NEJMra074111 18272895 PMC2711392

[11] Broug-Holub E Toews GB van Iwaarden JF Strieter RM Kunkel SL Paine R III. Alveolar macrophages are required for protective pulmonary defenses in murine Klebsiella pneumonia: elimination of alveolar macrophages increases neutrophil recruitment but decreases bacterial clearance and survival. Infect Immun. (1997) 65:1139–46. 9119443 10.1128/iai.65.4.1139-1146.1997 PMC175109

[12] Nau GJ Richmond JF Schlesinger A Jennings EG Lander ES Young RA. Human macrophage activation programs induced by bacterial pathogens. Proc Natl Acad Sci USA. (2002) 99:1503–8. 10.1073/pnas.022649799 11805289 PMC122220

[13] Pittet LA Quinton LJ Yamamoto K Robson BE Ferrari JD Algul H. Earliest innate immune responses require macrophage RelA during pneumococcal pneumonia. Am J Respir Cell Mol Biol. (2011) 45:573–81. 10.1165/rcmb.2010-0210OC 21216972 PMC3175578

[14] Schuurs AH Verheul HA. Effects of gender and sex steroids on the immune response. J Steroid Biochem. (1990) 35:157–72. 10.1016/0022-4731(90)90270-3 2407902

[15] Verthelyi D. Sex hormones as immunomodulators in health and disease. Int Immunopharmacol. (2001) 1:983–93. 10.1016/s1567-5769(01)00044-3 11407317

[16] Perelman RH Palta M Kirby R Farrell PM. Discordance between male and female deaths due to the respiratory distress syndrome. Pediatrics. (1986) 78:238–44. 3737300

[17] Nielsen HC. Testosterone regulation of sex differences in fetal lung development. Proc Soc Exp Biol Med. (1992) 199:446–52. 10.3181/00379727-199-43379 1549623

[18] Weinstein Y Ran S Segal S. Sex-associated differences in the regulation of immune responses controlled by the MHC of the mouse. J Immunol. (1984) 132:656–61. 6228595

[19] Spitzer JA. Gender differences in some host defense mechanisms. Lupus. (1999) 8:380–3. 10.1177/096120339900800510 10455517

[20] FitzSimmons SC. The changing epidemiology of cystic fibrosis. J Pediatr. (1993) 122:1–9. 10.1016/s0022-3476(05)83478-x 8419592

[21] Kaplan V Angus DC Griffin MF Clermont G Scott Watson R Linde-Zwirble WT. Hospitalized community-acquired pneumonia in the elderly: age- and sex-related patterns of care and outcome in the United States. Am J Respir Crit Care Med. (2002) 165:766–72. 10.1164/ajrccm.165.6.2103038 11897642

[22] Caracta CF. Gender differences in pulmonary disease. Mt Sinai J Med. (2003) 70:215–24. 12968194

[23] Gannon CJ Pasquale M Tracy JK McCarter RJ Napolitano LM. Male gender is associated with increased risk for postinjury pneumonia. Shock. (2004) 21:410–4. 10.1097/00024382-200405000-00003 15087816

[24] Gutierrez F Masia M Mirete C Soldan B Rodriguez JC Padilla S. The influence of age and gender on the population-based incidence of community-acquired pneumonia caused by different microbial pathogens. J Infect. (2006) 53:166–74. 10.1016/j.jinf.2005.11.006 16375972

[25] Yamamoto Y Tomioka H Sato K Saito H Yamada Y Setogawa T. Sex differences in the susceptibility of mice to infection induced by *Mycobacterium intracellulare*. Am Rev Respir Dis. (1990) 142:430–3. 10.1164/ajrccm/142.2.430 2382907

[26] Yamamoto Y Saito H Setogawa T Tomioka H. Sex differences in host resistance to Mycobacterium marinum infection in mice. Infect Immun. (1991) 59:4089–96. 1937768 10.1128/iai.59.11.4089-4096.1991 PMC259001

[27] Yancey AL Watson HL Cartner SC Simecka JW. Gender is a major factor in determining the severity of mycoplasma respiratory disease in mice. Infect Immun. (2001) 69:2865–71. 10.1128/iai.69.5.2865-2871.2001 11292700 PMC98236

[28] Guilbault C Stotland P Lachance C Tam M Keller A Thompson-Snipes L. Influence of gender and interleukin-10 deficiency on the inflammatory response during lung infection with *Pseudomonas aeruginosa* in mice. Immunology. (2002) 107:297–305. 10.1046/j.1365-2567.2002.01508.x 12423305 PMC1782799

[29] Mikerov AN Gan X Umstead TM Miller L Chinchilli VM Phelps DS Sex differences in the impact of ozone on survival and alveolar macrophage function of mice after *Klebsiella pneumoniae* infection. Respir Res. (2008) 9:24 10.1186/1465-9921-9-24 18307797 PMC2268931

[30] Mikerov AN Haque R Gan X Guo X Phelps DS Floros J. Ablation of SP-A has a negative impact on the susceptibility of mice to *Klebsiella pneumoniae* infection after ozone exposure: sex differences. Respir Res. (2008) 9:77. 10.1186/1465-9921-9-77 19055785 PMC2655296

[31] Mikerov AN Hu S Durrani F Gan X Wang G Umstead TM. Impact of sex and ozone exposure on the course of pneumonia in wild type and SP-A (-/-) mice. Microb Pathog. (2012) 52:239–49. 10.1016/j.micpath.2012.01.005 22285567 PMC3608432

[32] Thorenoor N Umstead TM Zhang X Phelps DS Floros J. Survival of surfactant protein-A1 and SP-A2 transgenic mice after *Klebsiella pneumoniae* infection, exhibits sex-, gene-, and variant specific differences; treatment with surfactant protein improves survival. Front Immunol. (2018) 9:2404. 10.3389/fimmu.2018.02404 30459763 PMC6232836

[33] Durrani F Phelps DS Weisz J Silveyra P Hu S Mikerov AN. Gonadal hormones and oxidative stress interaction differentially affects survival of male and female mice after lung *Klebsiella pneumoniae* infection. Exp Lung Res. (2012) 38:165–72. 10.3109/01902148.2011.654045 22394250 PMC3651915

[34] Crouch E Hartshorn K Ofek I. Collectins and pulmonary innate immunity. Immunol Rev. (2000) 173:52–65. 10.1034/j.1600-065x.2000.917311.x 10719667

[35] Phelps DS. Surfactant regulation of host defense function in the lung: a question of balance. Pediatr Pathol Mol Med. (2001) 20:269–92. 11486734

[36] Wright JR Youmans DC. Pulmonary surfactant protein A stimulates chemotaxis of alveolar macrophage. Am J Physiol. (1993) 264(4 Pt 1):L338–44. 10.1152/ajplung.1993.264.4.L338 8476070

[37] Mariencheck WI Savov J Dong Q Tino MJ Wright JR. Surfactant protein A enhances alveolar macrophage phagocytosis of a live, mucoid strain of *P. aeruginosa*. Am J Physiol. (1999) 277:L777–86. 10.1152/ajplung.1999.277.4.L777 10516219

[38] Kremlev SG Umstead TM Phelps DS. Effects of surfactant protein A and surfactant lipids on lymphocyte proliferation *in vitro*. Am J Physiol. (1994) 267(4 Pt 1):L357–64. 10.1152/ajplung.1994.267.4.L357 7943339

[39] Borron P McCormack FX Elhalwagi BM Chroneos ZC Lewis JF Zhu S. Surfactant protein A inhibits T cell proliferation via its collagen-like tail and a 210-kDa receptor. Am J Physiol. (1998) 275:L679–86. 10.1152/ajplung.1998.275.4.L679 9755099

[40] Brinker KG Garner H Wright JR. Surfactant protein A modulates the differentiation of murine bone marrow-derived dendritic cells. Am J Physiol Lung Cell Mol Physiol. (2003) 284:L232–41. 10.1152/ajplung.00187.2002 12388334

[41] Floros J Hoover RR. Genetics of the hydrophilic surfactant proteins A and D. Biochim Biophys Acta. (1998) 1408:312–22. 10.1016/s0925-4439(98)00077-5 9813381

[42] Hoover RR Floros J. Organization of the human SP-A and SP-D loci at 10q22-q23. Physical and radiation hybrid mapping reveal gene order and orientation. Am J Respir Cell Mol Biol. (1998) 18:353–62. 10.1165/ajrcmb.18.3.3035 9490653

[43] Karinch AM Floros J. 5' splicing and allelic variants of the human pulmonary surfactant protein A genes. Am J Respir Cell Mol Biol. (1995) 12:77–88. 10.1165/ajrcmb.12.1.7811473 7811473

[44] DiAngelo S Lin Z Wang G Phillips S Ramet M Luo J. Novel, non-radioactive, simple and multiplex PCR-cRFLP methods for genotyping human SP-A and SP-D marker alleles. Dis Markers. (1999) 15:269–81. 10.1155/1999/961430 10689550 PMC3851098

[45] Oberley RE Snyder JM. Recombinant human SP-A1 and SP-A2 proteins have different carbohydrate-binding characteristics. Am J Physiol Lung Cell Mol Physiol. (2003) 284:L871–81. 10.1152/ajplung.00241.2002 12505869

[46] Wang G Bates-Kenney SR Tao JQ Phelps DS Floros J. Differences in biochemical properties and in biological function between human SP-A1 and SP-A2 variants, and the impact of ozone-induced oxidation. Biochemistry. (2004) 43:4227–39. 10.1021/bi036023i 15065867

[47] Mikerov AN Umstead TM Huang W Liu W Phelps DS Floros J. SP-A1 and SP-A2 variants differentially enhance association of *Pseudomonas aeruginosa* with rat alveolar macrophages. Am J Physiol Lung Cell Mol Physiol. (2005) 288:L150–8. 10.1152/ajplung.00135.2004 15377498

[48] Mikerov AN Wang G Umstead TM Zacharatos M Thomas NJ Phelps DS. Surfactant protein A2 (SP-A2) variants expressed in CHO cells stimulate phagocytosis of *Pseudomonas aeruginosa* more than do SP-A1 variants. Infect Immun. (2007) 75:1403–12. 10.1128/iai.01341-06 17220308 PMC1828577

[49] Karinch AM deMello DE Floros J. Effect of genotype on the levels of surfactant protein A mRNA and on the SP-A2 splice variants in adult humans. Biochem J. (1997) 321(Pt 1):39–47. 10.1042/bj3210039 9003399 PMC1218034

[50] Kumar AR Snyder JM. Differential regulation of SP-A1 and SP-A2 genes by cAMP, glucocorticoids, and insulin. Am J Physiol. (1998) 274:L177–85. 10.1152/ajplung.1998.274.2.L177 9486201

[51] Wang G Myers C Mikerov A Floros J. Effect of cysteine 85 on biochemical properties and biological function of human surfactant protein A variants. Biochemistry. (2007) 46:8425–35. 10.1021/bi7004569 17580966 PMC2531219

[52] Wang G Guo X Diangelo S Thomas NJ Floros J. Humanized SFTPA1 and SFTPA2 transgenic mice reveal functional divergence of SP-A1 and SP-A2: formation of tubular myelin *in-vivo* requires both gene products. J Biol Chem. (2010) 285:11998–2010. 10.1074/jbc.M109.046243 20048345 PMC2852938

[53] Silveyra P Raval M Simmons B Diangelo S Wang G Floros J. The untranslated exon B of human surfactant protein A2 mRNAs is an enhancer for transcription and translation. Am J Physiol Lung Cell Mol Physiol. (2011) 301:L795–803. 10.1152/ajplung.00439.2010 21840962 PMC3290452

[54] Silveyra P DiAngelo SL Floros J. An 11-nt sequence polymorphism at the 3'UTR of human SFTPA1 and SFTPA2 gene variants differentially affect gene expression levels and miRNA regulation in cell culture. Am J Physiol Lung Cell Mol Physiol. (2014) 307:L106–19. 10.1152/ajplung.00313.2013 24793167 PMC4080286

[55] Noutsios GT Ghattas P Bennett S Floros J. 14-3-3 isoforms bind directly exon B of the 5'-UTR of human surfactant protein A2 mRNA. Am J Physiol Lung Cell Mol Physiol. (2015) 309:L147–57. 10.1152/ajplung.00088.2015 26001776 PMC4504974

[56] Wang G Phelps DS Umstead TM Floros J Human SP-A protein variants derived from one or both genes stimulate TNF-alpha production in the THP-1 cell line. Am J Physiol Lung Cell Mol Physiol. (2000) 278:L946–54. 10.1152/ajplung.2000.278.5.L946 10781424

[57] Wang G Umstead TM Phelps DS Al-Mondhiry H Floros J. The effect of ozone exposure on the ability of human surfactant protein a variants to stimulate cytokine production. Environ Health Perspect. (2002) 110:79–84. 10.1289/ehp.0211079 11781168 PMC1240696

[58] Huang W Wang G Phelps DS Al-Mondhiry H Floros J. Human SP-A genetic variants and bleomycin-induced cytokine production by THP-1 cells: effect of ozone-induced SP-A oxidation. Am J Physiol Lung Cell Mol Physiol. (2004) 286:L546–53. 10.1152/ajplung.00267.2003 14617519

[59] Mikerov AN Umstead TM Gan X Huang W Guo X Wang G. Impact of ozone exposure on the phagocytic activity of human surfactant protein A (SP-A) and SP-A variants. Am J Physiol Lung Cell Mol Physiol. (2008) 294:L121–30. 10.1152/ajplung.00288.2007 17981957 PMC2964667

[60] Floros J Wang G Mikerov AN. Genetic complexity of the human innate host defense molecules, surfactant protein A1 (SP-A1) and SP-A2–impact on function. Crit Rev Eukaryot Gene Expr. (2009) 19:125–37. 10.1615/critreveukargeneexpr.v19.i2.30 19392648 PMC2967201

[61] Phelps DS Umstead TM Silveyra P Hu S Wang G Floros J. Differences in the alveolar macrophage proteome in transgenic mice expressing human SP-A1 and SP-A2. J Proteom Genom Res. (2013) 1:2–26. 10.14302/issn.2326-0793.jpgr-12-207 24729982 PMC3981560

[62] Phelps DS Umstead TM Floros J. Sex differences in the acute *in-vivo* effects of different human SP-A variants on the mouse alveolar macrophage proteome. J Proteomics. (2014) 108:427–44. 10.1016/j.jprot.2014.06.007 24954098 PMC4128237

[63] Tsotakos N Phelps DS Yengo CM Chinchilli VM Floros J. Single-cell analysis reveals differential regulation of the alveolar macrophage actin cytoskeleton by surfactant proteins A1 and A2: implications of sex and aging. Biol Sex Differ. (2016) 7:18. 10.1186/s13293-016-0071-0 26998217 PMC4797174

[64] Noutsios GT Thorenoor N Zhang X Phelps DS Umstead TM Durrani F. SP-A2 contributes to miRNA-mediated sex differences in response to oxidative stress: pro-inflammatory, anti-apoptotic, and anti-oxidant pathways are involved. Biol Sex Differ. (2017) 8:37. 10.1186/s13293-017-0158-2 29202868 PMC5716385

[65] Noutsios GT Thorenoor N Zhang X Phelps DS Umstead TM Durrani F. Major effect of oxidative stress on the male, but not female, SP-A1 type II cell miRNome. Front Immunol. (2019) 10:1514. 10.3389/fimmu.2019.01514 31354704 PMC6635478

[66] Lopez-Rodriguez E Pascual A Arroyo R Floros J Perez-Gil J. Human pulmonary surfactant protein SP-A1 provides maximal efficiency of lung interfacial films. Biophys J. (2016) 111:524–36. 10.1016/j.bpj.2016.06.025 27508436 PMC4982931

[67] Guth AM Janssen WJ Bosio CM Crouch EC Henson PM Dow SW. Lung environment determines unique phenotype of alveolar macrophages. Am J Physiol Lung Cell Mol Physiol. (2009) 296:L936–46. 10.1152/ajplung.90625.2008 19304907 PMC2692811

[68] Beharka AA Gaynor CD Kang BK Voelker DR McCormack FX Schlesinger LS. Pulmonary surfactant protein A up-regulates activity of the mannose receptor, a pattern recognition receptor expressed on human macrophages. J Immunol. (2002) 169:3565–73. 10.4049/jimmunol.169.7.3565 12244146

[69] Kuronuma K Sano H Kato K Kudo K Hyakushima N Yokota S. Pulmonary surfactant protein A augments the phagocytosis of Streptococcus pneumoniae by alveolar macrophages through a casein kinase 2-dependent increase of cell surface localization of scavenger receptor A. J Biol Chem. (2004) 279:21421–30. 10.1074/jbc.M312490200 14993215

[70] Gil M McCormack FX Levine AM. Surfactant protein A modulates cell surface expression of CR3 on alveolar macrophages and enhances CR3-mediated phagocytosis. J Biol Chem. (2009) 284:7495–504. 10.1074/jbc.M808643200 19155216 PMC2658045

[71] Phelps DS Umstead TM Floros J. Sex differences in the response of the alveolar macrophage proteome to treatment with exogenous surfactant protein-A. Proteome Sci. (2012) 10:44. 10.1186/1477-5956-10-44 22824420 PMC3570446

[72] Carey MA Card JW Voltz JW Germolec DR Korach KS Zeldin DC. The impact of sex and sex hormones on lung physiology and disease: lessons from animal studies. Am J Physiol Lung Cell Mol Physiol. (2007) 293:L272–278. 10.1152/ajplung.00174.2007 17575008

[73] Thorenoor N Zhang X Umstead TM Scott Halstead E Phelps DS Floros J. Differential effects of innate immune variants of surfactant protein-A1 (SFTPA1) and SP-A2 (SFTPA2) in airway function after *Klebsiella pneumoniae* infection and sex differences. Respir Res. (2018) 19:23. 10.1186/s12931-018-0723-1 29394894 PMC5797374

[74] Falagas ME Mourtzoukou EG Vardakas KZ. Sex differences in the incidence and severity of respiratory tract infections. Respir Med. (2007) 101:1845–63. 10.1016/j.rmed.2007.04.011 17544265

[75] de Torres JP Cote CG Lopez MV Casanova C Diaz O Marin JM. Sex differences in mortality in patients with COPD. Eur Respir J. (2009) 33:528–35. 10.1183/09031936.00096108 19047315

[76] Thorenoor N Kawasawa YI Gandhi CK Zhang X Floros J. Differential Impact of Co-expressed SP-A1/SP-A2 Protein on AM miRNome; Sex Differences. Front Immunol. (2019) 10:1960. 10.3389/fimmu.2019.01960 31475015 PMC6707024

[77] Allen IC. Bacteria-mediated acute lung inflammation. Methods Mol Biol. (2013) 1031:163–75. 10.1007/978-1-62703-481-4_19 23824899

[78] Robinson MD McCarthy DJ Smyth GK. edgeR: a Bioconductor package for differential expression analysis of digital gene expression data. Bioinformatics. (2010) 26:139–40. 10.1093/bioinformatics/btp616 19910308 PMC2796818

[79] Sun J Nishiyama T Shimizu K Kadota K. TCC: an R package for comparing tag count data with robust normalization strategies. BMC Bioinformatics. (2013) 14:219. 10.1186/1471-2105-14-219 23837715 PMC3716788

[80] Belperio JA Keane MP Burdick MD Londhe V Xue YY Li K. Critical role for CXCR2 and CXCR2 ligands during the pathogenesis of ventilator-induced lung injury. J Clin Invest. (2002) 110:1703–16. 10.1172/jci15849 12464676 PMC151632

[81] Sue RD Belperio JA Burdick MD Murray LA Xue YY Dy MC. CXCR2 is critical to hyperoxia-induced lung injury. J Immunol. (2004) 172:3860–8. 10.4049/jimmunol.172.6.3860 15004193

[82] Grigoryev DN Finigan JH Hassoun P Garcia JG. Science review: searching for gene candidates in acute lung injury. Crit Care. (2004) 8:440–7. 10.1186/cc2901 15566614 PMC1065043

[83] Jeyaseelan S Chu HW Young SK Worthen GS. Transcriptional profiling of lipopolysaccharide-induced acute lung injury. Infect Immun. (2004) 72:7247–56. 10.1128/iai.72.12.7247-7256.2004 15557650 PMC529166

[84] Mauney CH Hollis T. SAMHD1: Recurring roles in cell cycle, viral restriction, cancer, and innate immunity. Autoimmunity. (2018) 51:96–110. 10.1080/08916934.2018.1454912 29583030 PMC6117824

[85] Takeda K Akira S. STAT family of transcription factors in cytokine-mediated biological responses. Cytokine Growth Factor Rev. (2000) 11:199–207. 10.1016/s1359-6101(00)00005-8 10817963

[86] Darnell JEJr Kerr IM Stark GR. Jak-STAT pathways and transcriptional activation in response to IFNs and other extracellular signaling proteins. Science. (1994) 264:1415–21. 10.1126/science.8197455 8197455

[87] Israel A. The IKK complex, a central regulator of NF-kappaB activation. Cold Spring Harb Perspect Biol. (2010) 2:a000158. 10.1101/cshperspect.a000158 20300203 PMC2829958

[88] Sun L Deng L Ea CK Xia ZP Chen ZJ. The TRAF6 ubiquitin ligase and TAK1 kinase mediate IKK activation by BCL10 and MALT1 in T lymphocytes. Mol Cell. (2004) 14:289–301. 10.1016/s1097-2765(04)00236-9 15125833

[89] Koptides M Umstead TM Floros J Phelps DS. Surfactant protein A activates NF-kappa B in the THP-1 monocytic cell line. Am J Physiol. (1997) 273(2 Pt 1):L382–8. 10.1152/ajplung.1997.273.2.L382 9277450

[90] Wang G Umstead TM Hu S Mikerov AN Phelps DS Floros J. Differential effects of human SP-A1 and SP-A2 on the BAL proteome and signaling pathways in response to *Klebsiella pneumoniae* and ozone exposure. Front Immunol. (2019) 10:561. 10.3389/fimmu.2019.00561 30972061 PMC6443908

[91] Janic B Umstead TM Phelps DS Floros J. Modulatory effects of ozone on THP-1 cells in response to SP-A stimulation. Am J Physiol Lung Cell Mol Physiol. (2005) 288:L317–25. 10.1152/ajplung.00125.2004 15466251

[92] Haque R Umstead TM Ponnuru P Guo X Hawgood S Phelps DS. Role of surfactant protein-A (SP-A) in lung injury in response to acute ozone exposure of SP-A deficient mice. Toxicol Appl Pharmacol. (2007) 220:72–82. 10.1016/j.taap.2006.12.017 17307210 PMC1906716

[93] Song M Phelps DS. Interaction of surfactant protein A with lipopolysaccharide and regulation of inflammatory cytokines in the THP-1 monocytic cell line. Infect Immun. (2000) 68:6611–7. 10.1128/iai.68.12.6611-6617.2000 11083772 PMC97757

[94] Mages J Dietrich H Lang R. A genome-wide analysis of LPS tolerance in macrophages. Immunobiology. (2007) 212:723–37. 10.1016/j.imbio.2007.09.015 18086374

[95] Nguyen HA Rajaram MV Meyer DA Schlesinger LS. Pulmonary surfactant protein A and surfactant lipids upregulate IRAK-M, a negative regulator of TLR-mediated inflammation in human macrophages. Am J Physiol Lung Cell Mol Physiol. (2012) 303:L608–16. 10.1152/ajplung.00067.2012 22886503 PMC3469587

[96] Gu J Zhou Y Huang L Ou W Wu J Li S. TP53 mutation is associated with a poor clinical outcome for non-small cell lung cancer: evidence from a meta-analysis. Mol Clin Oncol. (2016) 5:705–13. 10.3892/mco.2016.1057 28101350 PMC5228103

[97] Seifart C Lin HM Seifart U Plagens A DiAngelo S von Wichert P. Rare SP-A alleles and the SP-A1-6A(4) allele associate with risk for lung carcinoma. Clin Genet. (2005) 68:128–36. 10.1111/j.1399-0004.2005.00470.x 15996209

[98] Lin Z Thomas NJ Bibikova M Seifart C Wang Y Guo X. DNA methylation markers of surfactant proteins in lung cancer. Int J Oncol. (2007) 31:181–91. 10.3892/ijo.31.1.181 17549420

[99] Grageda M Silveyra P Thomas NJ DiAngelo SL Floros J. DNA methylation profile and expression of surfactant protein A2 gene in lung cancer. Exp Lung Res. (2015) 41:93–102. 10.3109/01902148.2014.976298 25514367 PMC4336205

[100] Russo P Tomino C Santoro A Prinzi G Proietti S Kisialiou A FKBP5 rs4713916: a potential genetic predictor of interindividual different response to inhaled corticosteroids in patients with chronic obstructive pulmonary disease in a real-life setting. Int J Mol Sci. (2019) 20:2024 10.3390/ijms20082024 PMC6514776 31022961

[101] Riley CM Sciurba FC. Diagnosis and outpatient management of chronic obstructive pulmonary disease: a review. JAMA. (2019) 321:786–97. 10.1001/jama.2019.0131 30806700

[102] Bari MR Hiron MM Zaman SM Rahman MM Ganguly KC. Microbes responsible for acute exacerbation of COPD. Mymensingh Med J. (2010) 19:576–85. 20956903

[103] Flattet Y Garin N Serratrice J Perrier A Stirnemann J Carballo S. Determining prognosis in acute exacerbation of COPD. Int J Chron Obstruct Pulmon Dis. (2017) 12:467–75. 10.2147/copd.S122382 28203070 PMC5293360

[104] Guo X Lin HM Lin Z Montano M Sansores R Wang G. Surfactant protein gene A, B, and D marker alleles in chronic obstructive pulmonary disease of a Mexican population. Eur Respir J. (2001) 18:482–90. 10.1183/09031936.01.00043401 11589345

[105] Mitsuhashi A Goto H Kuramoto T Tabata S Yukishige S Abe S. Surfactant protein A suppresses lung cancer progression by regulating the polarization of tumor-associated macrophages. Am J Pathol. (2013) 182:1843–53. 10.1016/j.ajpath.2013.01.030 23499372 PMC4429178

[106] Gomes M Teixeira AL Coelho A Araujo A Medeiros R. The role of inflammation in lung cancer. Adv Exp Med Biol. (2014) 816:1–23. 10.1007/978-3-0348-0837-8_1 24818717

[107] Bierne H Cossart P. When bacteria target the nucleus: the emerging family of nucleomodulins. Cell Microbiol. (2012) 14:622–33. 10.1111/j.1462-5822.2012.01758.x 22289128

[108] Areschoug T Gordon S. Scavenger receptors: role in innate immunity and microbial pathogenesis. Cell Microbiol. (2009) 11:1160–9. 10.1111/j.1462-5822.2009.01326.x 19388903

[109] Arredouani M Yang Z Ning Y Qin G Soininen R Tryggvason K. The scavenger receptor MARCO is required for lung defense against pneumococcal pneumonia and inhaled particles. J Exp Med. (2004) 200:267–72. 10.1084/jem.20040731 15263032 PMC2212010

[110] Sun K Metzger DW. Inhibition of pulmonary antibacterial defense by interferon-gamma during recovery from influenza infection. Nat Med. (2008) 14:558–64. 10.1038/nm1765 18438414

[111] Momburg F Hammerling GJ. Generation and TAP-mediated transport of peptides for major histocompatibility complex class I molecules. Adv Immunol. (1998) 68:191–256. 10.1016/s0065-2776(08)60560-x 9505090

[112] Pfeifer JD Wick MJ Roberts RL Findlay K Normark SJ Harding CV. Phagocytic processing of bacterial antigens for class I MHC presentation to T cells. Nature. (1993) 361:359–62. 10.1038/361359a0 7678924

[113] Vitalis TZ Zhang QJ Alimonti J Chen SS Basha G Moise A. Using the TAP component of the antigen-processing machinery as a molecular adjuvant. PLoS Pathog. (2005) 1:e36. 10.1371/journal.ppat.0010036 16389301 PMC1323471

[114] Dozmorov M Wu W Chakrabarty K Booth JL Hurst RE Coggeshall KM. Gene expression profiling of human alveolar macrophages infected by *B. anthracis* spores demonstrates TNF-alpha and NF-kappab are key components of the innate immune response to the pathogen. BMC Infect Dis. (2009) 9:152. 10.1186/1471-2334-9-152 19744333 PMC2752459

[115] Willsey GG Ventrone S Schutz KC Wallace AM Ribis JW Suratt BT. Pulmonary surfactant promotes virulence gene expression and biofilm formation in *Klebsiella pneumoniae*. Infect Immun. (2018) 86:18. 10.1128/iai.00135-18 29712730 PMC6013664

[116] Koussounadis A Langdon SP Um IH Harrison DJ Smith VA. Relationship between differentially expressed mRNA and mRNA-protein correlations in a xenograft model system. Sci Rep. (2015) 5:10775. 10.1038/srep10775 26053859 PMC4459080

[117] Tagaram HR Wang G Umstead TM Mikerov AN Thomas NJ Graff GR. Characterization of a human surfactant protein A1 (SP-A1) gene-specific antibody; SP-A1 content variation among individuals of varying age and pulmonary health. Am J Physiol Lung Cell Mol Physiol. (2007) 292:L1052–63. 10.1152/ajplung.00249.2006 17189324

[118] Wang Y Voelker DR Lugogo NL Wang G Floros J Ingram JL. Surfactant protein A is defective in abrogating inflammation in asthma. Am J Physiol Lung Cell Mol Physiol. (2011) 301:L598–606. 10.1152/ajplung.00381.2010 21784968 PMC3191759

[119] Eisenhauer PB Lehrer RI. Mouse neutrophils lack defensins. Infect Immun. (1992) 60:3446–7. 1639513 10.1128/iai.60.8.3446-3447.1992 PMC257335

[120] Haley PJ. Species differences in the structure and function of the immune system. Toxicology. (2003) 188:49–71. 10.1016/s0300-483x(03)00043-x 12748041

[121] Takeuchi O Akira S. Pattern recognition receptors and inflammation. Cell. (2010) 140:805–20. 10.1016/j.cell.2010.01.022 20303872

[122] Hajjar AM Ernst RK Tsai JH Wilson CB Miller SI. Human Toll-like receptor 4 recognizes host-specific LPS modifications. Nat Immunol. (2002) 3:354–9. 10.1038/ni777 11912497

[123] Montminy SW Khan N McGrath S Walkowicz MJ Sharp F Conlon JE. Virulence factors of Yersinia pestis are overcome by a strong lipopolysaccharide response. Nat Immunol. (2006) 7:1066–73. 10.1038/ni1386 16980981

[124] Mikerov AN Phelps DS Gan X Umstead TM Haque R Wang G. Effect of ozone exposure and infection on bronchoalveolar lavage: sex differences in response patterns. Toxicol Lett. (2014) 230:333–44. 10.1016/j.toxlet.2014.04.008 24769259 PMC4169765

[125] Mizgerd JP Skerrett SJ. Animal models of human pneumonia. Am J Physiol Lung Cell Mol Physiol. (2008) 294:L387–98. 10.1152/ajplung.00330.2007 18162603

[126] D'Ovidio F Floros J Aramini B Lederer D DiAngelo SL Arcasoy S. Donor surfactant protein A2 polymorphism and lung transplant survival. Eur Respir J. (2020) 55. 10.1183/13993003.00618-2019 31831583

[127] Nalian A Umstead TM Yang CH Silveyra P Thomas NJ Floros J. Structural and functional determinants of rodent and human surfactant protein A: a synthesis of binding and computational data. Front Immunol. (2019) 10:2613. 10.3389/fimmu.2019.02613 31781112 PMC6856657

[128] Garcia-Verdugo I Wang G Floros J Casals C Structural analysis and lipid-binding properties of recombinant human surfactant protein a derived from one or both genes. Biochemistry. (2002) 41:14041–53. 10.1021/bi026540l 12437362

