# Supplementary material for: Sex-Specific Regulation of Gene Expression Networks by Surfactant Protein A (SP-A) Variants in Alveolar Macrophages in Response to Klebsiella pneumoniae
Source: Front Immunol. 2020 Jun 24;11:1290. doi: 10.3389/fimmu.2020.01290 (PMC7326812; doi:10.3389/fimmu.2020.01290)
Supplement: Supplementary Figure 1 — Venn diagrams show the number of genes identified in comparisons of combined males and females from mice each carrying a different human variant in response to K. pneumoniae. (A) Comparison between SP-A1 (6A2 vs. 6A4). Out of 196 and 494 genes identified from 6A2 and 6A4 respectively, 5 are identified to be in common in both the SP-A1 gene-specific variants, 191 are specific to 6A2, and 489 are to 6A4. (B) Comparison between SP-A2 (1A0 vs. 1A3). Out of 276 and 397 genes identified from 1A0 and 1A3, respectively, 31 are identified to be in common in both the SP-A2 gene-specific variants, 245 are specific to 1A0 and 366 are to 1A3. (C) Comparison of genes between SP-A2 (1A0) vs. SP-A1 (6A2). Out of 276 and 196 genes identified from 1A0 and 6A2, respectively, 10 are identified to be in common in the SP-A1 (6A2) and SP-A2 (1A0) gene variants, 266 are specific to 1A0 and 186 are to 6A2. (D) Comparison of genes between SP-A2 (1A0) vs. SP-A1 (6A4). Out of 276 and 494 genes identified from 1A0 and 6A4, respectively, 15 are identified to be in common in the SP-A1 (6A4) and SP-A2 (1A0) gene variants, 261 are specific to 1A0 and 479 are to 6A4. (E) Comparison of genes between SP-A2 (1A3) vs. SP-A1 (6A2). Out of 397 and 196 genes identified from 1A3 and 6A2, respectively, 14 are identified to be in common in the SP-A1 (6A2) and SP-A2 (1A3) gene variants, 384 are specific to 1A3 and 182 are to 6A2. (F) Comparison of genes between SP-A2 (1A3) vs. SP-A1 (6A4). Out of 397 and 494 genes identified from 1A3 and 6A4, respectively, 31 are identified to be in common in the SP-A1 (6A4) and SP-A2 (1A3) gene variants, 366 are specific to 1A3 and 463 are to 6A4. [file Image_1.pdf]

Supplementary Figure 1

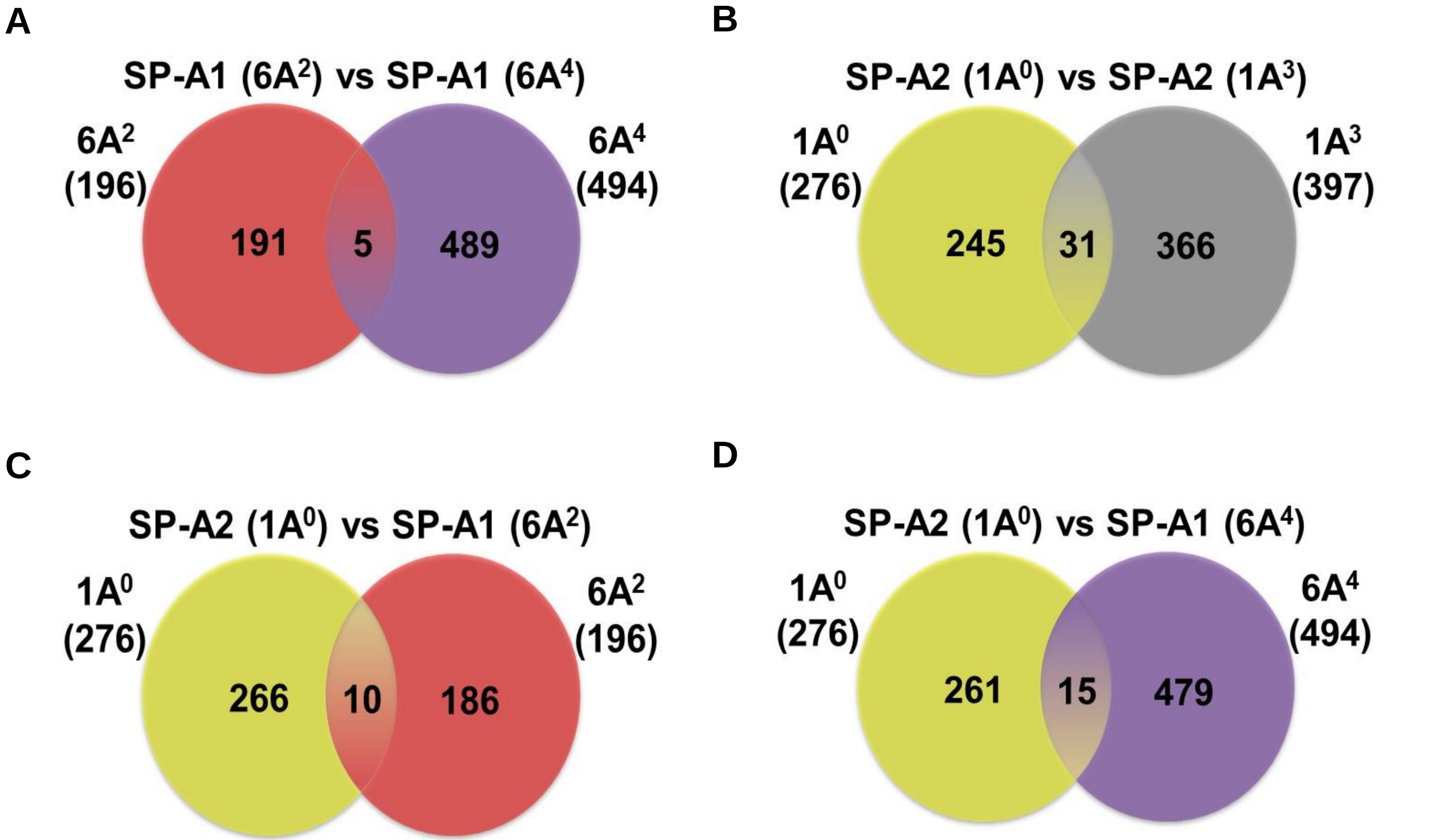

Supplementary Figure 1

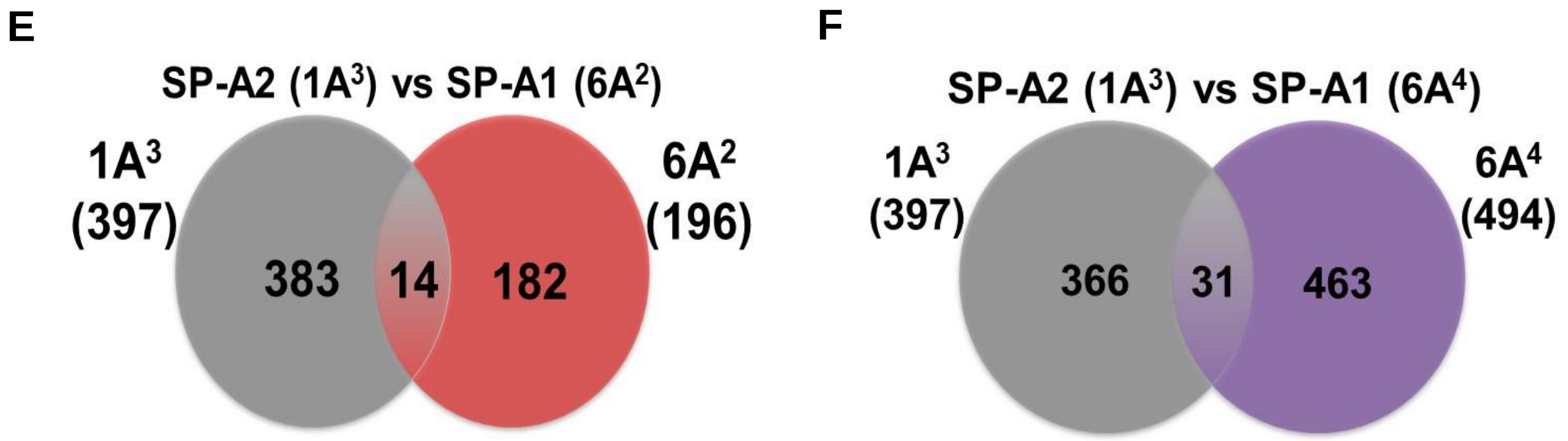

In all panels males and females are combined.
