# Supplementary figures and images for: Sex-Specific Regulation of Gene Expression Networks by Surfactant Protein A (SP-A) Variants in Alveolar Macrophages in Response to Klebsiella pneumoniae
Source: Front Immunol. 2020 Jun 24;11:1290. doi: 10.3389/fimmu.2020.01290 (PMC7326812; doi:10.3389/fimmu.2020.01290)

**Supplementary Figure 2**

**A**

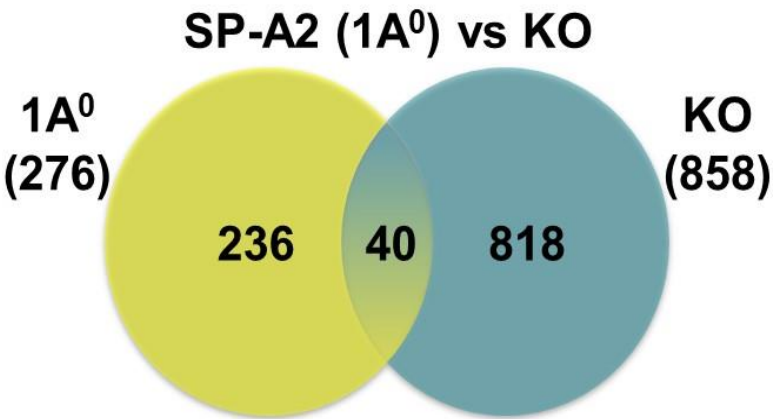

**B**

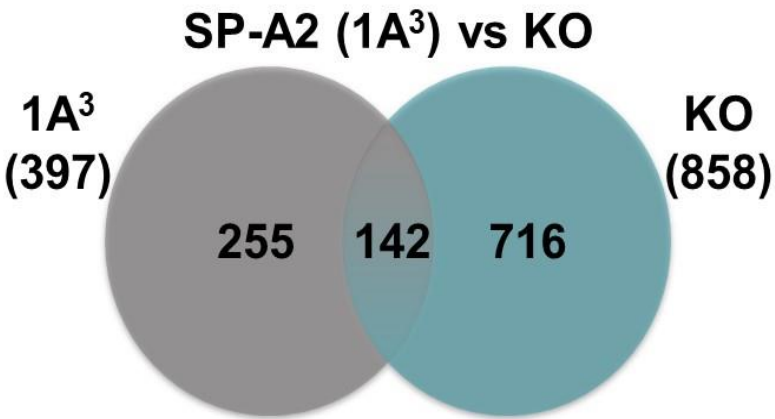

**C**

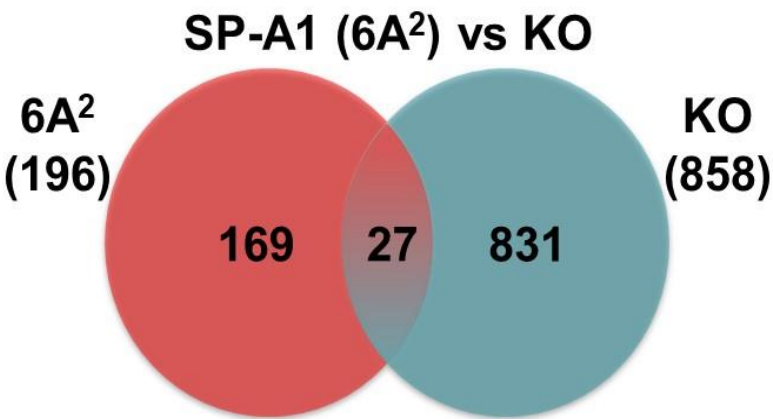

**D**

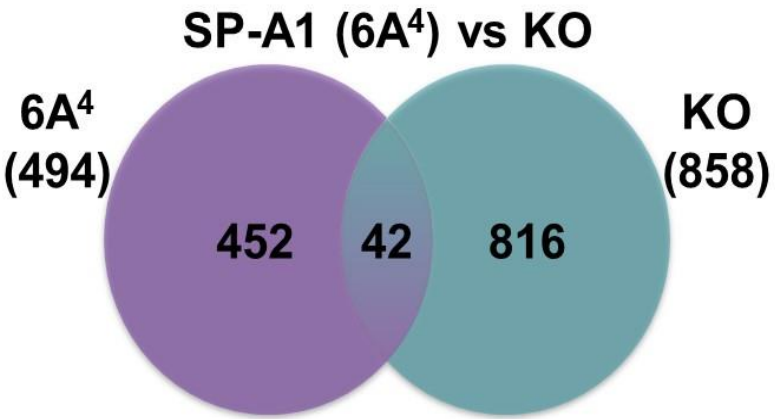

**In all panels males and females are combined.**

Supplement: Supplementary Figure 2 — Venn diagram comparisons of the number of genes from different variants vs. KO. (A) Comparison between SP-A2 (1A0) vs. KO. Out of 276 and 858 genes identified from 1A0 and KO, respectively, 40 are identified to be in common in the SP-A2 (1A0) and KO, 236 are specific to 1A0 and 818 are to KO. (B) Comparison between SP-A2 (1A3) vs. KO. Out of 397 and 858 genes identified from 1A3 and KO, respectively, 142 are identified to be in common in the SP-A2 (1A3) and KO, 255 are specific to 1A3 and 716 are to KO. (C) Comparison between SP-A1 (6A2) vs. KO. Out of 196 and 858 genes identified from 6A2 and KO, respectively, 27 are identified to be in common in the SP-A1 (6A2) and KO, 169 are specific to 6A2 and 831 are to KO. (D) Comparison between SP-A1 (6A4) vs. KO. Out of 494 and 858 genes identified from 6A4 and KO, respectively, 42 are identified to be in common in the SP-A1 (6A4) and KO, 452 are specific to 6A4 and 816 are to KO. [file Image_2.pdf]
