# Supplementary material for: Sex-Specific Regulation of Gene Expression Networks by Surfactant Protein A (SP-A) Variants in Alveolar Macrophages in Response to Klebsiella pneumoniae
Source: Front Immunol. 2020 Jun 24;11:1290. doi: 10.3389/fimmu.2020.01290 (PMC7326812; doi:10.3389/fimmu.2020.01290)

Supplementary Figure 3

SP-A1 (6A^2)

Males

Cell cycle signaling node-1

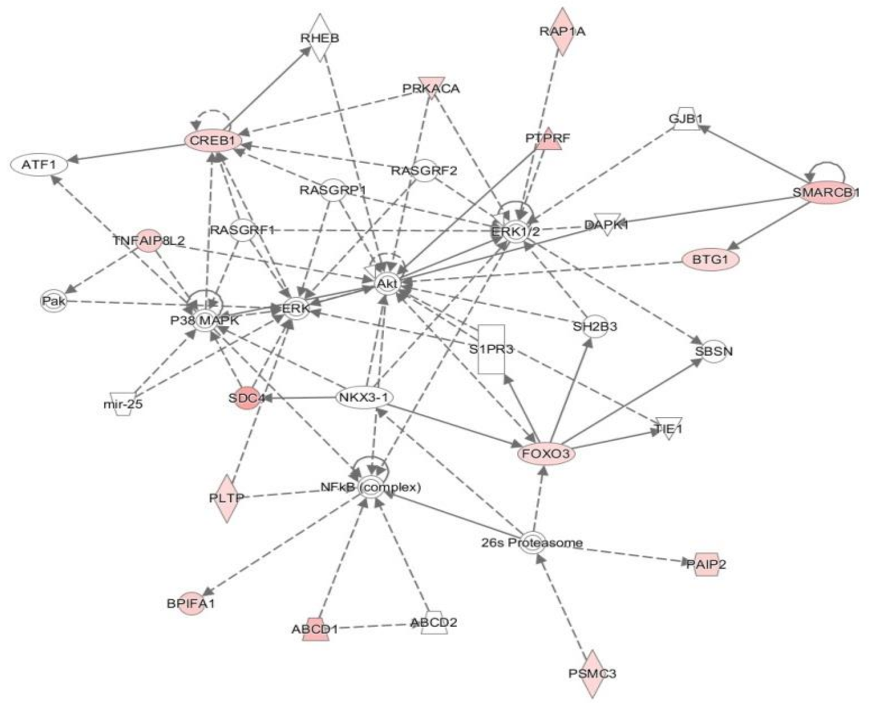

Cell cycle signaling node-2

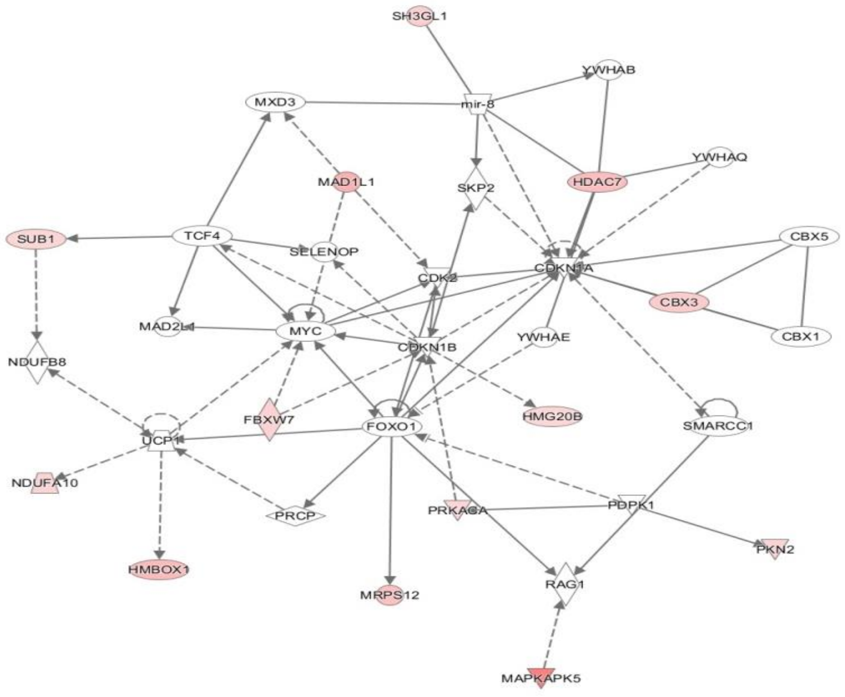

TP-53 node

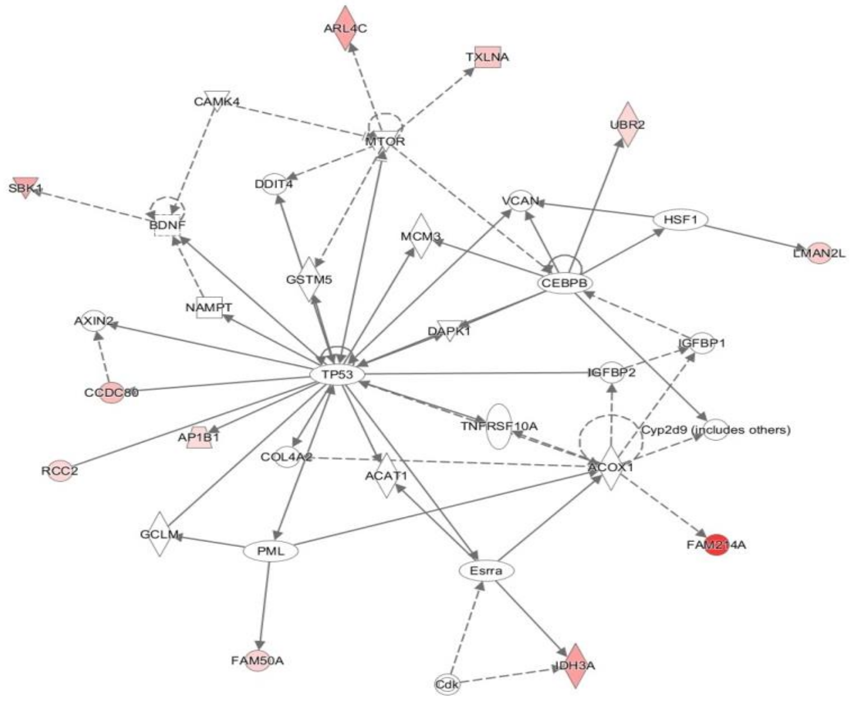

Females

TP-53 node

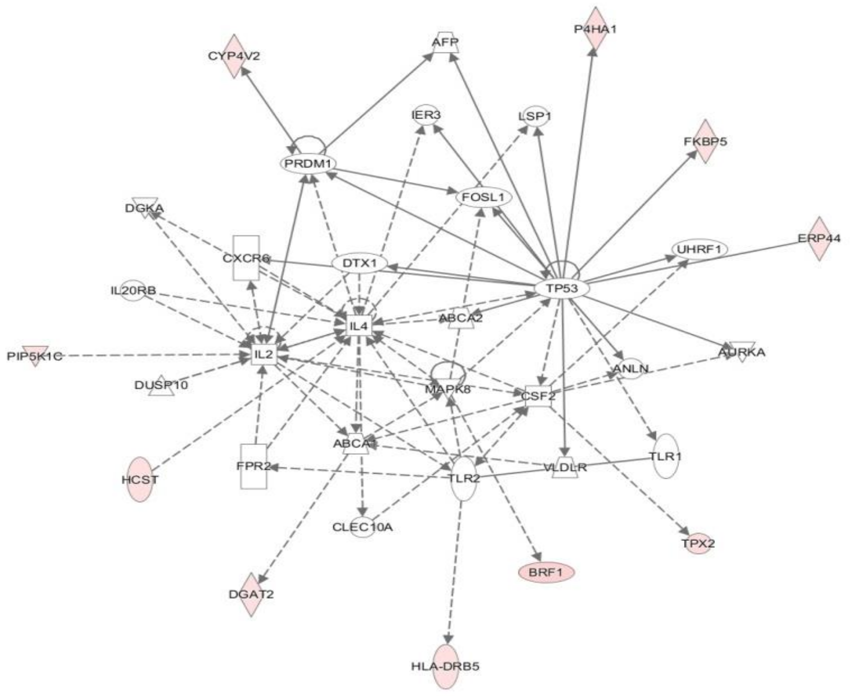

SP-A1 (6A[4])

Males

TP-53 node

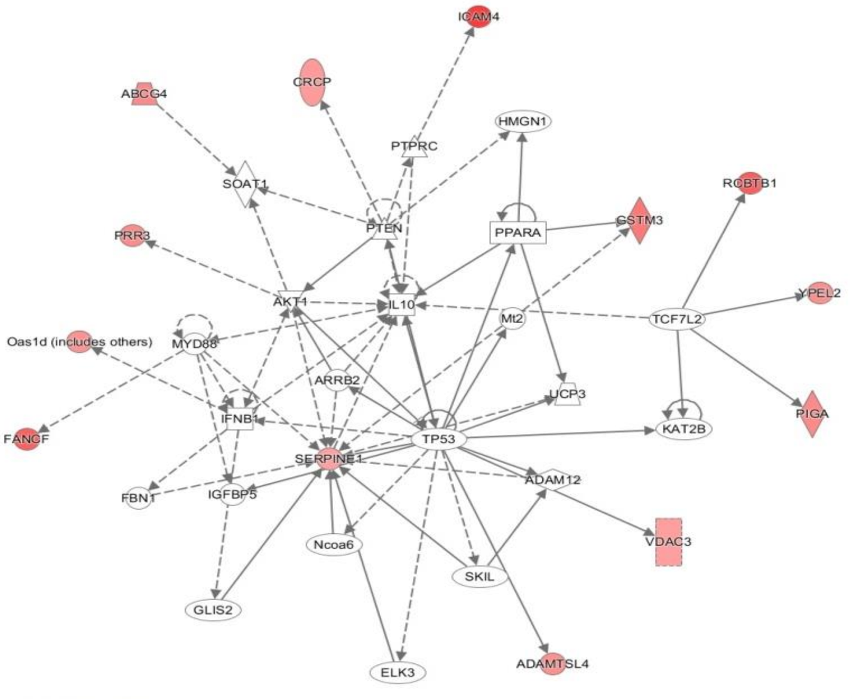

Females

TP-53 node

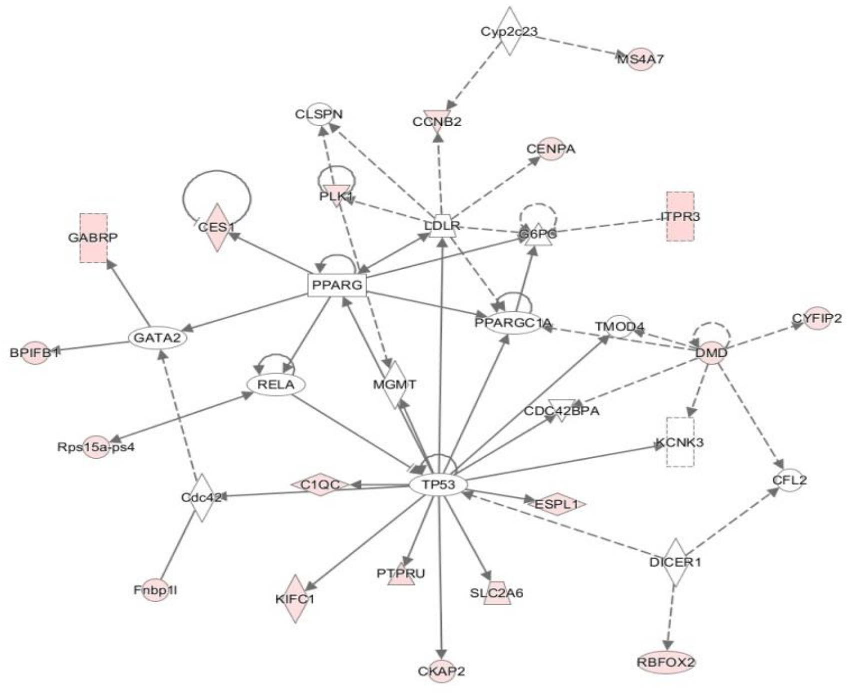

SP-A2 (1A[3])

Females

Cell cycle signaling node

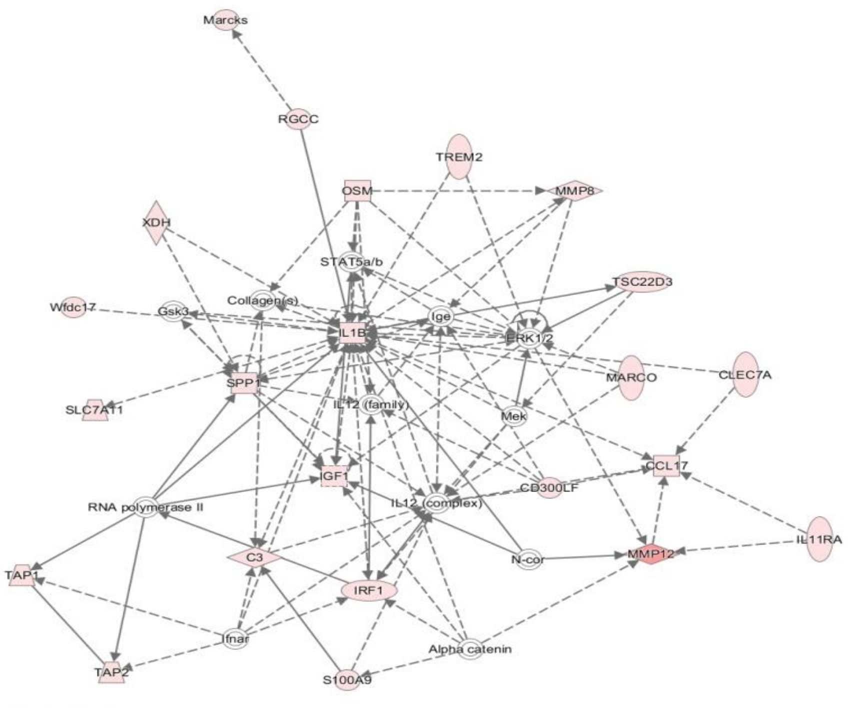

KO

Males | **Females**

TNF node

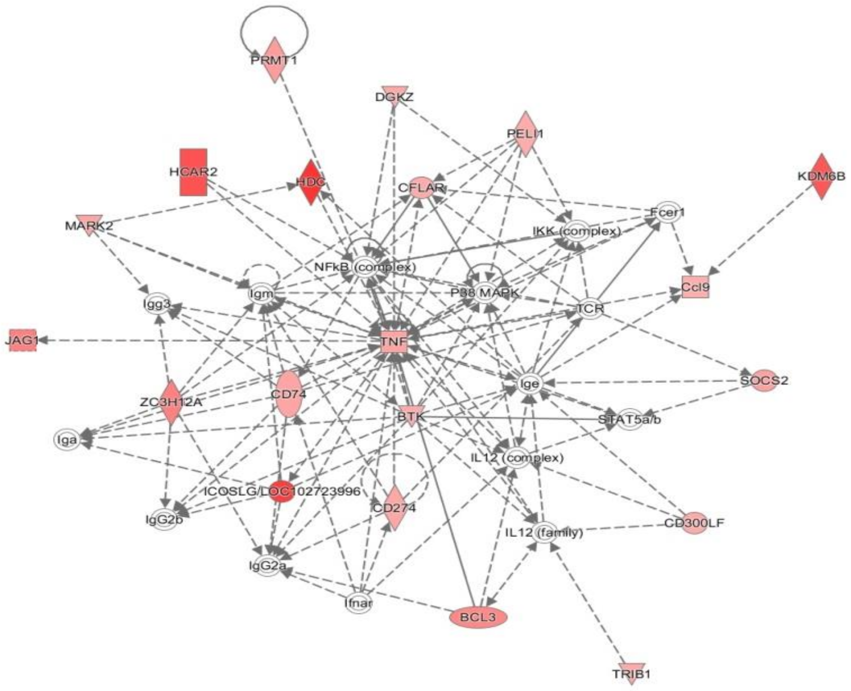

TP-53 node

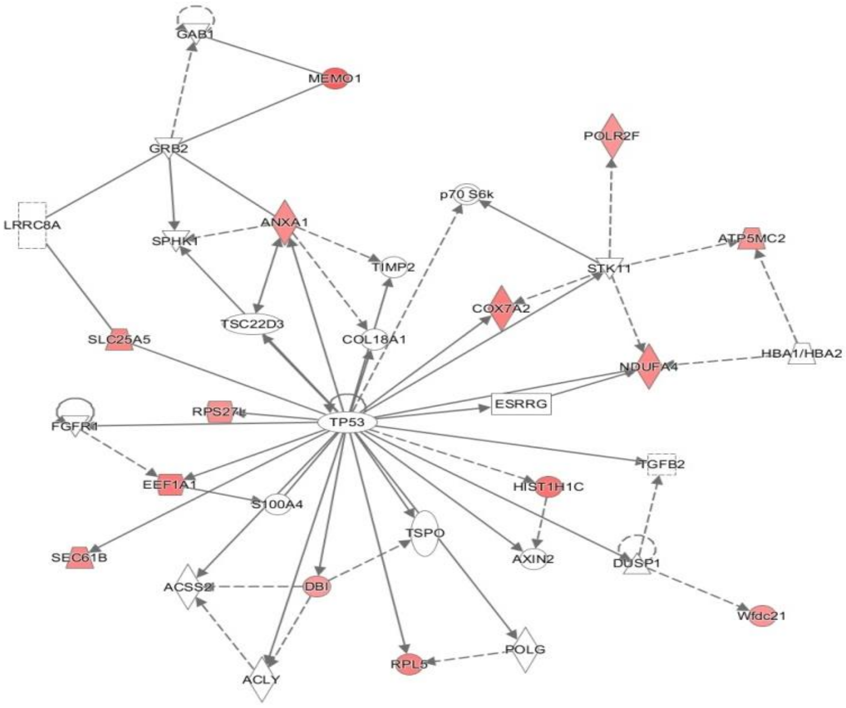

Cell cycle signaling node

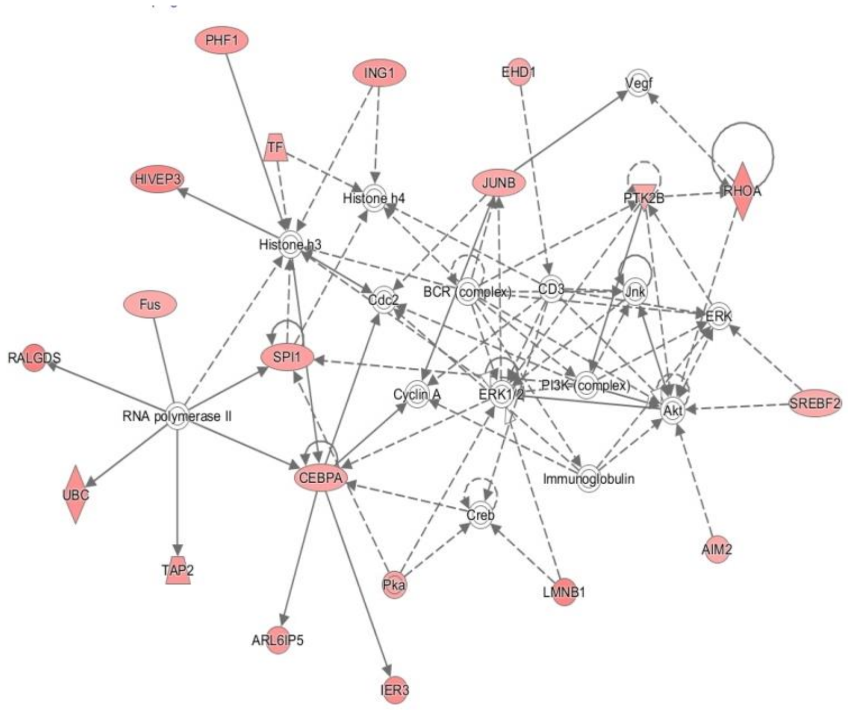

TP-53 node

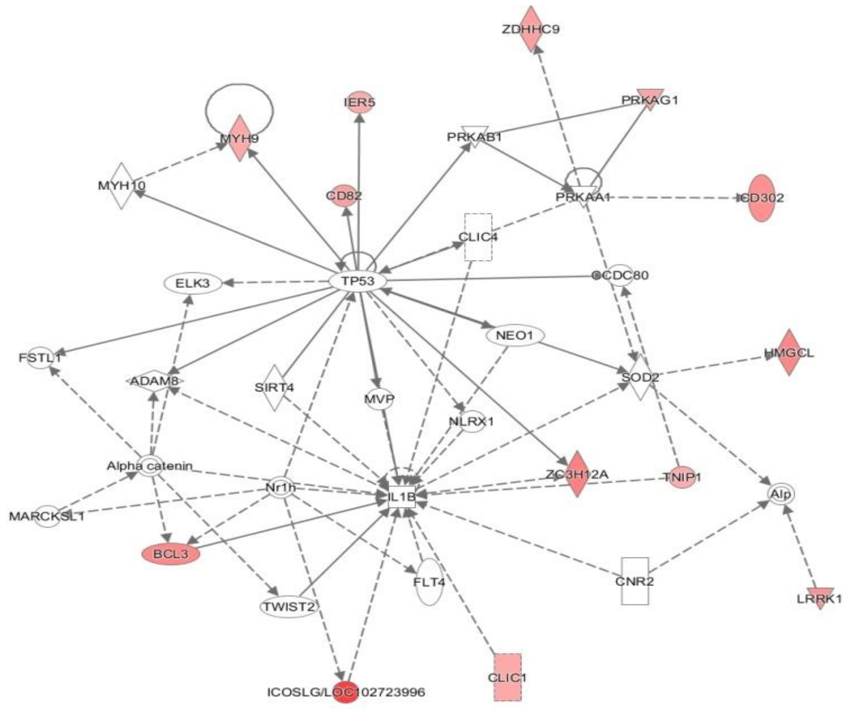

Supplement: Supplementary Figure 3 — Ingenuity Pathway Analysis for gene expression in males and females exposed to K. pneumoniae infection for 6 h in SP-A1 (6A2, 6A4), SP-A2 (1A3) and KO. Diagrams of biological networks of selected genes whose expression was ≥2-fold in the AM of male and female mice after infection are shown in color. Left, genes and pathways in male mice; right, genes and pathways in female mice. The diagrams show reported direct (solid lines) or indirect (dashed lines) gene interactions. Each gene or group of genes is represented as a node. Molecules that are significantly altered ≥2 are represented as node in red. Node shapes represent functional classes of gene products: Square for cytokines, Concentric (double) circle for complex/group, Diamonds for enzymes and peptidases, Ovals for transcription regulators and Transmembrane receptors, Triangle for phosphatases and Kinases, Rectangles for ligand-dependent nuclear receptors, G-protein coupled receptors, and ion channels, Trapezoids for transporters and microRNAs. [file Image_3.pdf]
